# Person-Generated Health Data in Women’s Health: Scoping Review

**DOI:** 10.2196/53327

**Published:** 2024-05-16

**Authors:** Jalisa Lynn Karim, Rachel Wan, Rhea S Tabet, Derek S Chiu, Aline Talhouk

**Affiliations:** 1 Department of Obstetrics and Gynaecology University of British Columbia Vancouver, BC Canada; 2 Department of Pharmacology and Therapeutics McGill University Montréal, QC Canada; 3 Department of Molecular Oncology University of British Columbia Vancouver, BC Canada

**Keywords:** digital health, women’s health, mobile health, health app, wearables, femtech, self-tracking, personalized health, person-generated health data, patient-generated health data, scoping review, mobile phone

## Abstract

**Background:**

The increased pervasiveness of digital health technology is producing large amounts of person-generated health data (PGHD). These data can empower people to monitor their health to promote prevention and management of disease. Women make up one of the largest groups of consumers of digital self-tracking technology.

**Objective:**

In this scoping review, we aimed to (1) identify the different areas of women’s health monitored using PGHD from connected health devices, (2) explore personal metrics collected through these technologies, and (3) synthesize facilitators of and barriers to women’s adoption and use of connected health devices.

**Methods:**

Following the PRISMA (Preferred Reporting Items for Systematic Reviews and Meta-Analyses) guidelines for scoping reviews, we searched 5 databases for articles published between January 1, 2015, and February 29, 2020. Papers were included if they targeted women or female individuals and incorporated digital health tools that collected PGHD outside a clinical setting.

**Results:**

We included a total of 406 papers in this review. Articles on the use of PGHD for women steadily increased from 2015 to 2020. The health areas that the articles focused on spanned several topics, with pregnancy and the postpartum period being the most prevalent followed by cancer. Types of digital health used to collect PGHD included mobile apps, wearables, websites, the Internet of Things or smart devices, 2-way messaging, interactive voice response, and implantable devices. A thematic analysis of 41.4% (168/406) of the papers revealed 6 themes regarding facilitators of and barriers to women’s use of digital health technology for collecting PGHD: (1) accessibility and connectivity, (2) design and functionality, (3) accuracy and credibility, (4) audience and adoption, (5) impact on community and health service, and (6) impact on health and behavior.

**Conclusions:**

Leading up to the COVID-19 pandemic, the adoption of digital health tools to address women’s health concerns was on a steady rise. The prominence of tools related to pregnancy and the postpartum period reflects the strong focus on reproductive health in women’s health research and highlights opportunities for digital technology development in other women’s health topics. Digital health technology was most acceptable when it was relevant to the target audience, was seen as user-friendly, and considered women’s personalization preferences while also ensuring accuracy of measurements and credibility of information. The integration of digital technologies into clinical care will continue to evolve, and factors such as liability and health care provider workload need to be considered. While acknowledging the diversity of individual needs, the use of PGHD can positively impact the self-care management of numerous women’s health journeys. The COVID-19 pandemic has ushered in increased adoption and acceptance of digital health technology. This study could serve as a baseline comparison for how this field has evolved as a result.

**International Registered Report Identifier (IRRID):**

RR2-10.2196/26110

## Introduction

### Background

The practice of keeping notes to monitor one’s health is not a recent phenomenon. Individuals have long recognized the benefits of tracking various health aspects, including the ability to be more active participants in managing their health, gaining a more complete picture of their health, and reducing the frequency of in-person appointments; however, this tracking was previously done through paper logs [1]. Today, with the proliferation of digital tools, self-tracking has significantly evolved and become more prevalent. The increasing pervasiveness of technology, particularly mobile phones, has seamlessly integrated it into our daily lives, making self-tracking more accessible and convenient than ever before [2]. Connected digital health technologies such as smartphones, wearables (eg, smartwatches), sensors, the Internet of Things (eg, internet-enabled weight scales), and web-based applications have permeated society and are increasingly adopted to collect and track health data. In 2021, a total of 87% of Canadians owned a smartphone, up by 73% from 2009 [3]. With >350,000 digital health apps accessible via these smartphones [4], approximately two-thirds of Canadians digitally track at least one aspect of their health [5]; similar statistics have been reported in the United States [6]. Moreover, since the introduction and popularization of fitness trackers in 2010, sensors and wearable devices have increasingly become part of daily life [2]. During the global COVID-19 pandemic, self-tracking took on even greater significance [7,8]. With the heightened awareness of health and the need for proactive measures, individuals have turned to self-tracking to monitor their well-being and make informed decisions. With this transformation, self-tracking has transcended its previous boundaries, offering individuals new opportunities to optimize their well-being and ushering in a new era of personalized health care [9-11].

Digital health tools have revolutionized the active and passive collection of health data through various applications and wearable devices. These various digital health tools collect and generate an unprecedented amount of data that can be used to glean insights into one’s health. Person-generated health data (PGHD), which are clinically relevant data captured outside traditional care settings [12], provide valuable insights that empower users to self-monitor and reflect on their health. PGHD can refer to any data collected from wearable and smart devices as well as self-input information into platforms such as mobile apps and websites. By leveraging digital technologies, individuals can collect and store their health data, enabling them to actively manage their own health and monitor chronic conditions. Furthermore, the integration of these data with research presents an opportunity to improve the patients’ experience and enhance personalized medicine. The recognition of this opportunity has started to take shape with patient-reported outcome measures and patient-reported experience measures being increasingly recognized as essential information to assess quality of care and prioritize patient-centered approaches and with mandatory assessment as part of clinical trials [13]. Seamlessly linking PGHD that are captured outside traditional care settings with clinical data and disease models can unlock new possibilities for tailored treatments and predictive informatics. The integration of digital health tools not only facilitates patient-provider communication but also offers opportunities for education, increased awareness, self-tracking, and self-monitoring without burdening health care resources. By focusing on the individual’s experience, personalization, and prevention, digital health tools contribute to a patient-centered care paradigm that aims to optimize health care outcomes and improve overall well-being while empowering patients to take charge of their health.

In recent years, the emergence of femtech, defined as technology-driven solutions specifically designed to address women’s health needs and concerns, has revolutionized the landscape of self-tracking and health care for women [14]. Femtech encompasses a wide range of digital tools, such as period-tracking apps, fertility monitors, pregnancy trackers, and menopause management platforms. These innovative solutions empower women to track and manage their reproductive health, menstrual cycles, and overall well-being with greater accuracy and ease. Femtech has not only provided women with personalized insights into their bodies but has also helped break taboos and encouraged open conversations about topics that were once stigmatized or ignored. The rapid growth of femtech has promoted access to women’s health information, greater autonomy in decision-making, and enhanced overall health care experiences for women worldwide. It has become an integral part of the self-tracking movement, demonstrating the transformative power of technology in promoting women’s health and well-being.

### Objectives

In this study, we reviewed the use of digital tools and PGHD in women’s health research, focusing on articles published between January 1, 2015, and February 29, 2020, before the COVID-19 pandemic. Our review encompassed various connected health devices, which included both passive data collection devices such as wearable sensors and active input devices such as smartphone apps and websites. This review sought to accomplish the following:

1. Identify the different areas of women’s health and health-related behaviors monitored using PGHD from connected health devices.
2. Explore personal metrics collected through these technologies.
3. Synthesize facilitators and barriers that impact women’s adoption and use of connected health devices in managing their health.

## Methods

### Overview

This scoping review was conducted based on our previously published protocol [15]. We adopted the PRISMA-ScR (Preferred Reporting Items for Systematic Reviews and Meta-Analyses extension for Scoping Reviews) guidelines [16]. The completed checklist is provided in Multimedia Appendix 1 [16].

### Search Strategy

The search strategy was designed in close collaboration with a reference librarian with input from the authors (JLK and AT). We searched a total of 5 databases: MEDLINE, Embase, APA PsycINFO, CINAHL Complete, and Web of Science Core Collection. Initial searches were completed in early March 2020. Searches were limited to articles published in 2015 or later because publications with the keyword “digital health” started to emerge in the literature around that time [17], and with the fast evolution of the field, previous articles may not be relevant to the current landscape. Keywords and subject headings were designed to search the literature for the intersection of the following 4 topics: women, health, digital devices, and tracking. The full search strategy, including a full list of search terms, was published with the protocol [15] and is available in Multimedia Appendix 2.

### Eligibility Criteria

We were interested in digital technologies and interventions targeting women and people assigned female at birth. To be included in the review, studies needed to specifically target women, focus on female-only health topics (eg, menstruation), or only include female participants. We included a variety of publication types but excluded conference abstracts and conference reviews, editorials, letters, and comments due to the limited details in such literature.

We excluded articles that presented digital health tools designed for health care providers as we were primarily interested in devices and apps that women can engage with outside a clinical setting. Articles only discussing the use of real-time consultations, whether through video, phone, or web-based chat, were excluded. We excluded articles that described digital health tools used solely for educational purposes; to maintain the focus of the review on tracking or monitoring one’s data for health, devices must have allowed users to input personal health data.

The complete inclusion and exclusion criteria are presented in Textbox 1. We decided to retain the original inclusion end date of February 29, 2020, to maintain a focus on the literature before the COVID-19 pandemic and avoid potential complexities caused by pandemic-related disruptions in research and health care practices. Concentrating on prepandemic literature also established a clear baseline for future comparisons and allowed us to maintain feasibility of completion without compromising quality given the broad scope of the review.

**Inclusion and exclusion criteria.**

**Inclusion criteria**

- Published between January 1, 2015, and February 29, 2020
- Refers to a health issue that pertains only to women or comprises only female participants of any age
- Includes the use of connected health tools for tracking or monitoring some aspect of health, which could include smartphone apps, wearable devices, the Internet of Things (eg, Bluetooth- or internet-enabled glucometers, blood pressure cuffs, and weight scales), and implantable devices
- Involves data collection from the user of the connected health tool (ie, the user either manually inputs data into the device or they are automatically uploaded)
- The user must be able to interact with the app or device on her own at home (outside a clinical setting)
- Available in English

**Exclusion criteria**

- Not available in English
- Conference abstracts, conference reviews, editorials, letters, or comments
- Study media releases and user reviews of specific applications
- Research conducted on animals
- Research involving male participants
- Tracking of infants and children unless tracking breastfeeding (because breastfeeding is directly related to the mother’s health and body)
- Devices or apps that are meant for health care provider use or use in a clinical setting only or cannot be used independently without a health care provider present
- Digital health tools that are only for educational or informational purposes and do not allow the user to enter or track her own data (ie, no information exchange)
- Telemedicine services (eg, live video consultations with health care providers)

### Study Selection

We imported the results from the database searches to the Covidence systematic review software (Veritas Health Innovation). Covidence detected records believed to be duplicates, and these were manually checked before removing them. In addition, some articles were manually recognized as duplicates during the screening process and were subsequently tagged as duplicates and removed. Screening was conducted independently by at least 2 reviewers (JLK, RST, and AT) at both the abstract screening stage and the full-text screening stage. We attempted to contact the corresponding authors of articles that passed abstract screening when we were unable to locate the full text. Conflicts at either stage were discussed and agreed upon among the 3 authors involved in the screening process.

### Data Charting and Deviations From the Protocol

The final list of data charting elements is provided in Textbox 2. Data charting for all elements except for usability and acceptability was conducted using Google Sheets created by the study team. The categories for different data charting options were initially created based on a small subset of articles and were discussed among the authors involved in the charting process. The team met regularly throughout the data charting process to discuss and refine coding categories that best summarized the data. Starting with more granular categories and later combining them into broader concepts was necessary to summarize the number of articles included in this review. For each article included, data were charted by one reviewer (RW or RST) and verified for accuracy by a second reviewer (JLK). Data were summarized in bar graphs, maps, and tables (JLK, RST, and DSC), as presented in the following sections. For the locations, we recorded the countries from which the participants were recruited (if applicable). If an article did not describe recruiting participants, then the countries of the authors were recorded based on the authors’ affiliations.

**Data charting elements.**

**Article information**

- Title
- Authors
- Year of first publication

**Study characteristics**

- Country or countries in which the research was conducted
- Research study type

**Contexts for women’s connected health**

- Health areas of focus

**Digital device details**

- Types of digital health
- Metrics collected by the devices

**Usability and acceptability**

- Facilitators of and barriers to the use of the technologies (coded into themes)

For the thematic analysis, articles that mentioned any aspect of usability, acceptability, facilitators, or barriers to the use of digital health tools were imported into NVivo (R1 2020; QSR International). Coding was done independently by 2 reviewers (JLK and RW) and then combined through discussions. As with the data charting process, we initially coded more granularly and then grouped the detailed codes together later in the analytic process. Decisions on how to group the codes into themes and subthemes were made through group consensus (JLK, RW, and AT).

In our protocol, we indicated that we would extract the name of the device or app used in each study. While we did complete this step in our data charting, we have not presented the results in this paper. Several articles either did not specify the brand name (eg, only specified that it was a mobile app) or had digital health tools named after the study, so we did not find this information useful to showcase in our results. There were no other deviations from the published protocol.

## Results

### Study Selection

**Figure 1:**
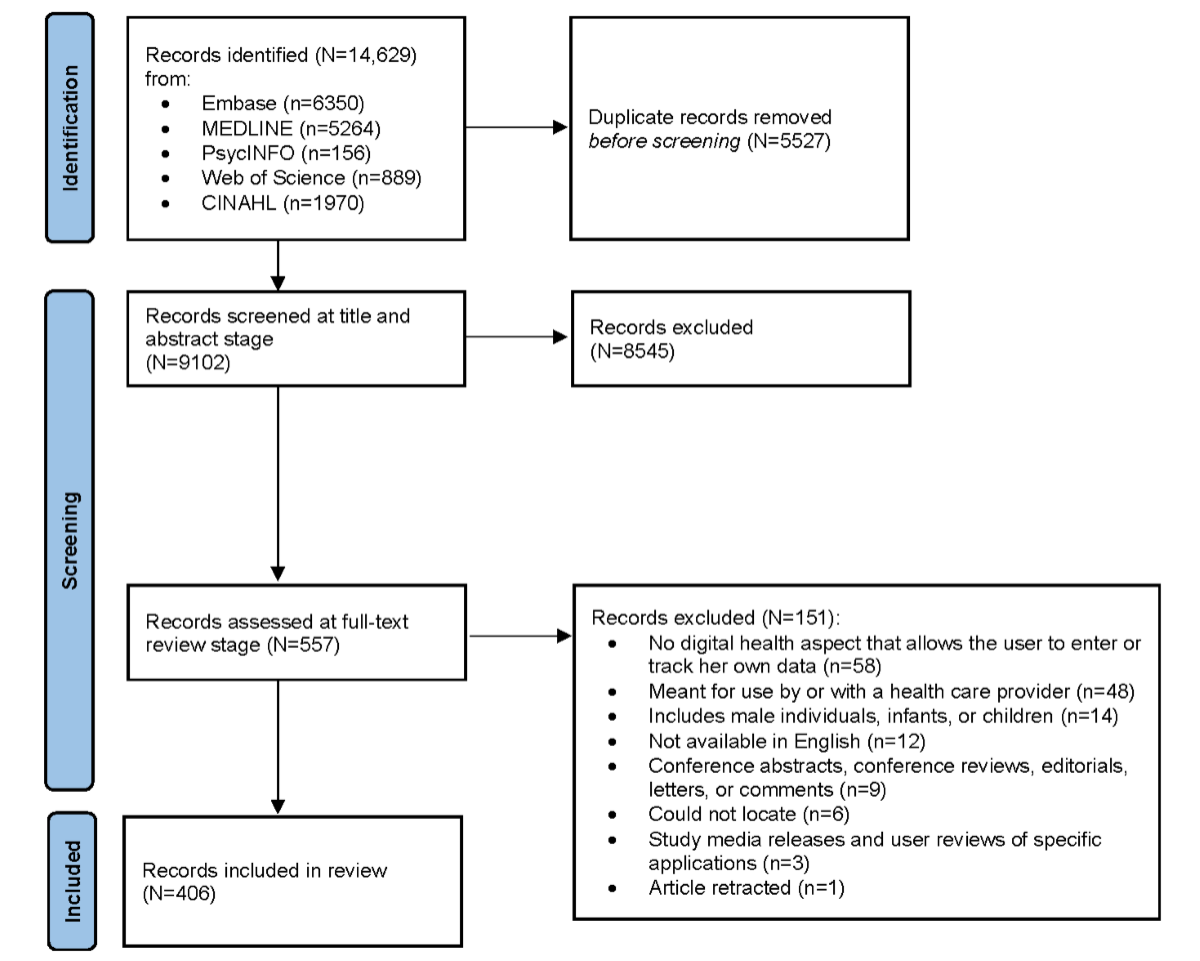
PRISMA (Preferred Reporting Items for Systematic Reviews and Meta-Analyses) flow diagram.

The searches identified 14,629 records that were imported into the Covidence software for deduplication and screening. After deduplication, a total of 9102 articles were screened for relevance, and 8545 (93.88%) were excluded based on title and abstract. From reading the full texts of the remaining 557 records, an additional 151 (27.1%) were excluded. The most common reasons for exclusion were the inability of study participants to enter or track their own data (58/151, 38.4%) or because the digital health technology was designed to be used by or with a health care provider (48/151, 31.8%). The remaining 406 publications were included in the scoping review. Some of the included publications reported on the same research project; in those cases, all of them were included. Our search did not encounter any articles that directly addressed or mentioned the inclusion of intersex, transgender, or nonbinary participants. The PRISMA (Preferred Reporting Items for Systematic Reviews and Meta-Analyses) flow diagram detailing the full study selection process is shown in Figure 1. The list of included articles sorted by health areas of focus can be found in Multimedia Appendix 3 [18-58].

### Year and Country

There was an increasing trend in number of publications per year, with 10.1% (41/406) of the articles published in 2015, a total of 13.3% (54/406) of the articles published in 2016, a total of 18% (73/406) of the articles published in 2017, a total of 26.4% (107/406) of the articles published in 2018, and 29.6% (120/406) of the articles published in 2019. Only 2.7% (11/406) of the publications were from 2020 because our cutoff date for inclusion was February 29, 2020.

**Figure 2:**
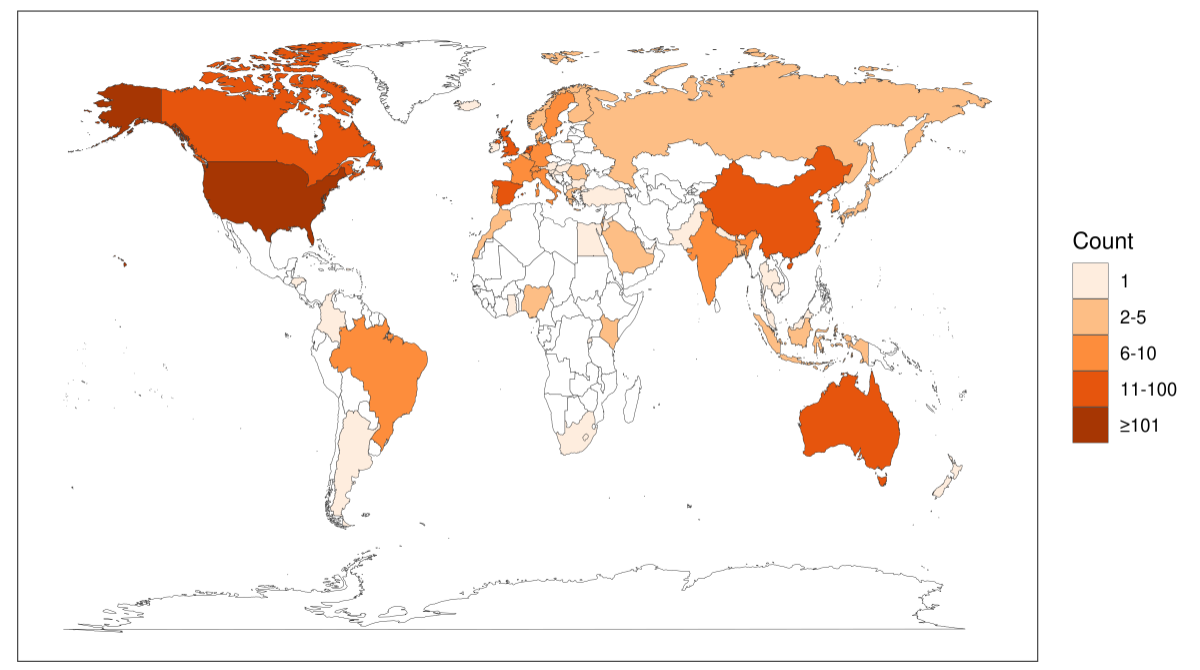
Number of publications by country.

Articles included in the review covered worldwide research, including every continent except Antarctica (Figure 2). As we only considered articles written in English, most of the articles were published in Western, English-speaking countries, primarily the United States (169/406, 41.6% of the articles), the United Kingdom (34/406, 8.4% of the articles), Australia (33/406, 8.1% of the articles), and Canada (19/406, 4.7% of the articles). Other countries where several included articles were published were China (13/406, 3.2% of the articles), the Netherlands (13/406, 3.2% of the articles), Spain (13/406, 3.2% of the articles), and Sweden (10/406, 2.5% of the articles).

Interestingly, of the 169 articles from the United States, 26 (15.4%) specifically focused on African American or Black, ethnic minority, or low-income women. One study from Singapore specifically included multiethnic women [18], and a study from Australia included Indigenous Australian women as their participants [19]. In addition, one review conducted by researchers in Australia looked specifically at studies with women from culturally and linguistically diverse backgrounds [20].

### Study Types

**Figure 3:**
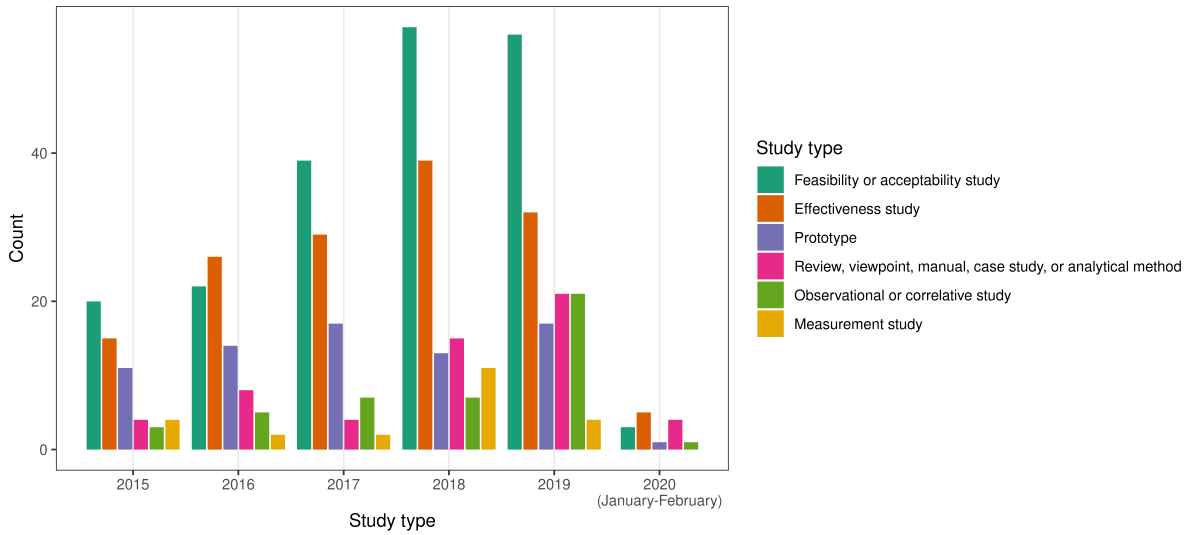
Study type by year of publication.

The types of studies that used digital health tools in women’s health research are reported in Figure 3 by year of publication (note that the articles could fall into more than one study category). The most common study type encountered was feasibility or acceptability studies (197/406, 48.5% of the articles, including 9/197, 4.6% protocols), followed by effectiveness studies (146/406, 36% of the articles, including 36/146, 24.7% protocols) and publications reporting on digital tool prototypes (73/406, 18% of the articles). Effectiveness studies reported on outcome measures of an intervention, including randomized and nonrandomized trials with one or more study arms. Reviews (of published literature, apps, or wearables), viewpoints, manuals, case studies, or analytical methods (56/406, 13.8% of the articles combined) were also encountered. Observational or correlative studies (44/406, 10.8% of the articles, including 3/44, 7% protocols) were studies that observed the health behaviors of individuals through digital health technologies without assessing the effectiveness of an intervention or analyzed associations between variables (eg, associations between heart rate and loss-of-control eating) [21]. Finally, measurement studies (23/406, 5.7% of the articles) reported on the validity, reliability, or accuracy of a digital health tool.

### Health Areas of Focus

The analysis of the reviewed articles highlighted research in several recurring women’s health areas of focus. A full breakdown of the health areas is reported in Table 1 (articles could fall into more than one health area). Pregnancy and the postpartum period emerged as the most prominent health area with 42.6% (173/406) of the articles. Within this category, there was a specific emphasis on general care and monitoring (45/173, 26% of the articles), physical activity and diet (34/173, 19.7% of the articles), and glucose monitoring (31/173, 17.9% of the articles). Cancer was identified as the second most common health area, with 19.5% (79/406) of the articles dedicated to its exploration. Specifically, a significant focus was observed on the relationship between cancer and cardiovascular health, with 47% (37/79) of the articles addressing this aspect. The impact of lifestyle on overall health and well-being was also addressed, with 14.3% (58/406) of the articles delving into physical activity, sedentary behavior, diet, weight, and obesity. Menstrual, sexual, and reproductive health were explored in 12.1% (49/406) of the articles to shed light on various aspects of women’s reproductive health and associated concerns, with 76% (37/49) focusing on menstrual cycle tracking or fertility monitoring. Furthermore, 9.9% (40/406) of the articles were dedicated to chronic conditions (such as urinary incontinence, osteoporosis, and diabetes) with the aim of enhancing understanding and developing interventions for individuals living with chronic health conditions. To accommodate articles that did not fit within the primary health areas, an *Other* category comprising 6.4% (26/406) of the articles was established. This category included articles on athlete monitoring (10/26, 38% of the articles), such as heart rate monitoring during sports tournaments; mental health and quality of life (9/26, 35% of the articles); gender-based violence (3/26, 12% of the articles); and more. Finally, a small subset of 0.5% (2/406) of the articles did not align with any specific health area; these included a publication reporting results from a survey on African American women’s willingness to participate in eHealth research [22] and a publication analyzing women’s interactions with digital health technologies [23]. These articles were included because, although they did not discuss a specific health area, they still focused on women’s use of digital health tools in general.

**Table 1:** Health areas of focus (N=406).

Health area of focus		Studies, n (%)
**Pregnancy and the postpartum period (n=173)**		
	General care and monitoring	45 (26)
	Physical activity, diet, gestational weight gain, postpartum weight retention, and weight management	34 (19.7)
	Glucose monitoring and diabetes, including gestational diabetes	31 (17.9)
	Blood pressure monitoring; pre-eclampsia; and hypertension, including gestational hypertension	22 (12.7)
	Fetal monitoring and contraction monitoring	17 (9.8)
	Depression, stress, self-efficacy, and mental well-being	11 (6.4)
	Breastfeeding	7 (4)
	Alcohol or tobacco use reduction	6 (3.5)
	Cesarean section	2 (1.2)
	HIV prevention and care	2 (1.2)
	Sleep	2 (1.2)
	Asthma	2 (1.2)
	Vaccinations	2 (1.2)
	Preterm delivery	1 (0.6)
	Miscarriage	1 (0.6)
	Postabortion care	1 (0.6)
	Environmental exposures	1 (0.6)
	Pregnancy research	1 (0.6)
**Cancer (n=79)**		
	Cardiovascular or cardiometabolic health, physical activity, sedentary behavior, diet, weight, and obesity	37 (46.8)
	Support, quality of life, self-efficacy, and other psychosocial measures	17 (21.5)
	Prevention and early detection	9 (11.4)
	Side effects from therapy and treatment and symptom management	7 (8.9)
	Adjuvant endocrine therapy and medication adherence	6 (7.6)
	Depression, stress, anxiety, and fear of cancer recurrence	4 (5.1)
	Physical therapy and rehabilitation	3 (3.8)
	Postoperative outcomes and care	3 (3.8)
	Sleep	2 (2.5)
	Cancer survivorship	1 (1.3)
	Treatment-induced menopausal symptoms	1 (1.3)
**Lifestyle (n=58)**		
	Physical activity, sedentary behavior, diet, weight, and obesity	50 (86.2)
	Sleep	9 (15.5)
	Alcohol dependency and problem drinking	2 (3.4)
	Smoking cessation	2 (3.4)
	Loss-of-control eating	1 (1.7)
	Iron intake	1 (1.7)
**Menstrual, sexual, and reproductive health (n=49)**		
	Menstrual cycle tracking and fertility monitoring	37 (75.5)
	Pregnancy planning	4 (8.2)
	Contraception	4 (8.2)
	Menstrual pain, heavy menstrual bleeding, and bleeding disorders	3 (6.1)
	Sexually transmitted infections	2 (4.1)
	Menopause	2 (4.1)
	PCOS^a^	2 (4.1)
	Premenstrual symptoms	1 (2)
**Chronic conditions (n=40)**		
	Urinary incontinence and pelvic muscle dysfunction	12 (30)
	Osteoarthritis, osteoporosis, and bone health	8 (20)
	CVD^b^ or CVD risk	7 (17.5)
	Diabetes	4 (10)
	Asthma	2 (5)
	COPD^c^	1 (2.5)
	SLE^d^	1 (2.5)
	IC^e^ or BPS^f^	1 (2.5)
	Epilepsy medication adherence	1 (2.5)
	Chronic widespread pain	1 (2.5)
	Atrial fibrillation	1 (2.5)
	Prehypertension	1 (2.5)
	ABL^g^	1 (2.5)
**Other (n=26)**		
	Athlete monitoring	10 (38.5)
	Depression, mood, stress, self-efficacy, and quality of life	9 (34.6)
	Domestic violence, intimate partner violence, and gender-based violence	3 (11.5)
	Environmental exposures and air pollution	2 (7.7)
	Sweat analysis	1 (3.8)
	Adolescent health research	1 (3.8)
	Skin characteristics	1 (3.8)
No specific health area		2 (0.5)

^a^PCOS: polycystic ovary syndrome.

^b^CVD: cardiovascular disease.

^c^COPD: chronic obstructive pulmonary disease.

^d^SLE: systemic lupus erythematosus.

^e^IC: interstitial cystitis.

^f^BPS: bladder pain syndrome.

^g^ABL: accidental bowel leakage.

**Figure 4:**
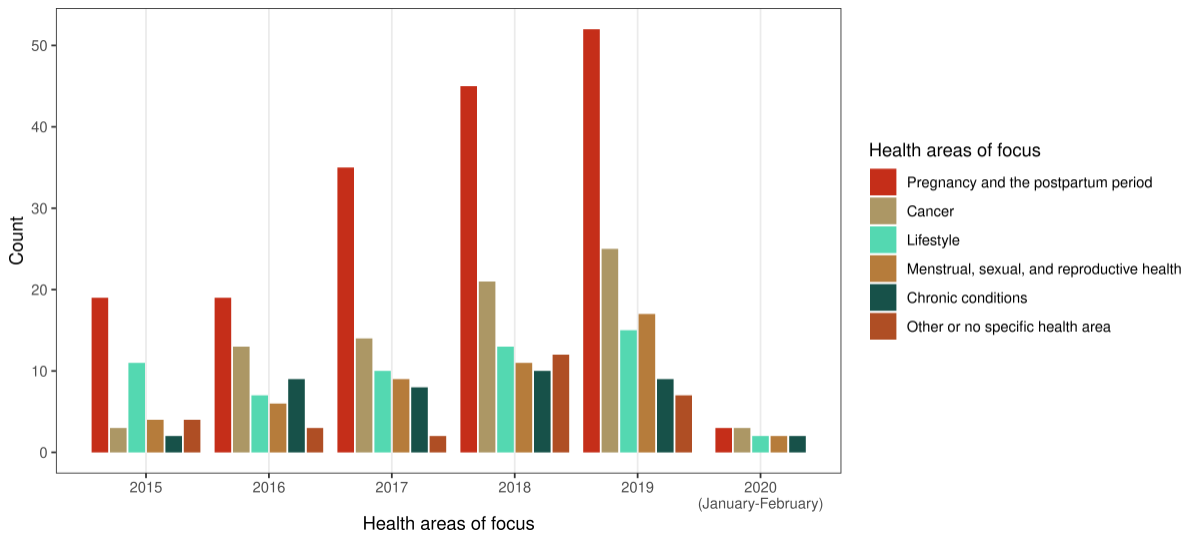
Health areas of focus by year of publication.

Figure 4 shows how the health areas of focus for women’s use of digital health changed over the years that were included in the review (2015-2019 plus January 2020-February 2020). There was an increasing trend from 2015 to 2020 in the number of publications focusing on pregnancy and the postpartum period, as well as cancer and menstrual, sexual, and reproductive health. However, articles focused on women’s use of digital health for lifestyle-related topics and chronic conditions did not see a notable increase over those years.

### Type of Digital Health and Metrics Collected

Within the articles reviewed, smartphone, mobile, or tablet apps emerged as the most prevalent type of digital health (295/406, 72.7% of the articles), followed by wearable devices (165/406, 40.6% of the articles) and websites or patient portals (93/406, 22.9% of the articles). Other types of technology were not investigated as much. For example, 13.5% (55/406) of the articles addressed smart devices or the Internet of Things (referring to objects with sensors that connect to a network, such as Bluetooth-enabled glucometers and blood pressure machines). Finally, 7.4% (30/406) of the articles reported on 2-way messaging, 1% (4/406) of the articles reported on interactive voice response telephone calls, and only 0.5% (2/406) of the articles reported on implantable devices. With respect to the metrics collected, we found >250 metrics, such as heart rate, number of steps, mood, ovulation test results, and days of menstruation. A full list of the metrics is reported in Multimedia Appendix 4.

### Thematic Analysis

#### Overview

Of the 406 articles included in this scoping review, 168 (41.4%) mentioned usability, acceptability, facilitators, or barriers to the use of digital health tools at least once. Our thematic analysis identified 6 themes: (1) accessibility and connectivity, (2) design and functionality, (3) accuracy and credibility, (4) audience and adoption, (5) impact on community and health service, and (6) impact on health and behavior. The themes are described in further detail in the following sections.

The thematic analysis detailed in the following sections is primarily based on the views of the participants in the studies we reviewed to provide a user perspective; however, one subsection in theme 5 focuses on the health care provider perspective.

#### Theme 1: Accessibility and Connectivity

The accessibility and connectivity of digital technologies emerged as an important theme with two subthemes: (1) cost and convenience and (2) connectivity, compatibility, and software issues.

##### Cost and Convenience

Our analysis revealed that the cost and convenience of digital tools collecting PGHD are important factors that can impact their adoption and use. On the one hand, digital health technologies can be seen as more affordable compared to traditional health care visits and more accessible to a wider range of people, including those of a lower socioeconomic status. On the other hand, they can also be perceived as too expensive and novelty items, and associated extra costs such as data plans can also be a barrier for some people. Because PGHD can be collected and entered throughout the day in real time, and because most people carry a phone around with them every day, these technologies offer greater convenience than traditional in-person health care encounters by providing anytime, anywhere virtual access and putting information at people’s fingertips through smartphones and web platforms. One user spoke about an in-app treatment program:

That was what was so good about this, I can do this at home myself, no need to book an appointment, find the time and suit others, and you know, that process of booking a time.

24

Some inconvenient aspects of digital health technologies include uncomfortable wearables that are too bulky, difficulty of use, or not fitting into the users’ lifestyles, as noted in one article:

Women also mentioned that the comfort of the wearable sensors was a barrier. Comfort became a barrier for some women during exercise and hot weather.

25

Devices with a short battery life and wearables that are not water resistant are also considered inconvenient as they require the user to frequently remember to charge the device or put the wearable back on after water-based activities. Certain restrictions, such as not being able to wear a device in a workplace, can also create inconvenient barriers for some users.

##### Connectivity, Compatibility, and Software Issues

Factors related to connectivity and other issues such as device synchronization, freezing, or disconnection can significantly impact the user experience and engagement with digital health tools. For example, the dependence on mobile and internet access can be a disadvantage. Cellphone and network coverage limitations can pose an important barrier in rural areas or during travel. Where mobile data or Wi-Fi connection are limited, people may struggle to use digital health tools that require internet connection; this can create disparities in access to health care resources, particularly for those of a lower socioeconomic status or living in remote communities with limited infrastructure. Incompatibility between operating systems such as Android and iOS, iPhone and iPad, or various browsers can also be an important barrier to accessing digital health technology.

Software issues can significantly impact the user experience of digital health technologies. Broken links can prevent users from accessing valuable information or features within apps or websites. App crashes can discourage users from engaging with the digital health tool altogether. In addition, slow loading times can negatively impact the user experience, making it challenging for users to access information or features quickly and efficiently.

#### Theme 2: Design and Functionality

The second theme centered on design and functionality and included four subthemes: (1) appearance and design; (2) functionality and features; (3) personalization; and (4) safety, privacy, and security.

##### Appearance and Design

Appearance and design play important roles in the success of connected health devices. In terms of app design, the color scheme and layout facilitate user-friendliness. Bad formatting can make it difficult for users to read or understand the content of an app or website. Font size that is too small can be challenging for those with visual impairments, and some color combinations can be difficult to read. The quality of the images used in digital health interventions can also impact user experience, with low-quality images potentially making it difficult for users to interpret the information being presented.

Apps that are visually appealing and easy to use are more likely to be successful. When it comes to wearables, women tend to prefer sleek, understated designs that are esthetically pleasing without being bulky. A sleek design can encourage use of the wearable. For example, some women consider their wearable to be a fashion item that sparks conversation, which encourages their continued use of the device, as illustrated in a participant quote:

Um, that it’s, like, kind of stylish, like, I feel, like, cool that I wear one. A lot of people ask me, they’re like, oh, which one is that, like, is that a Fitbit, is that an Apple watch? It has the interchangeable bands and stuff like that, so, you can, like, change the color of it and everything. It’s like a conversation piece.

26

Other women prefer more discretion in the design of wearables and their size or in the app icon on their mobile device because they do not want to reveal the purpose of the device to others. People may feel self-conscious when wearing the device or using the app, especially if it reveals their medical condition. For example, the following quote is from a study that incorporated a sensor band worn on the wrist to help female undergraduate students with problematic drinking:

P310 noted that while in class, “my professor commented on it which made me feel awkward.”

27

##### Functionality and Features

In terms of functionality, the availability of clinical interpretation of user data is deemed essential, and health warnings based on recorded PGHD are noted to be helpful. Moreover, notifications and reminders are also useful for improving adherence to self-tracking and maintaining goals, and users appreciate receiving automated SMS text messages and feedback on progress. Actionable advice is seen as very important, and women expressed a desire for more interaction and the ability to integrate with other apps. For example, users want the ability to access information from their health record and to be able to see graphical summaries of their data over time. Regarding the presentation of information, users appreciate concise information written in simple language. Choice of words is also perceived as especially important to ensure that the information is easy to understand. People enjoy the gamification of content, and the graphical presentation of results is found to be informative.

The ability to upload multimedia and the ability to customize the application’s displays and notifications are noted as features that improve user engagement and satisfaction. The ability to record voice notes and consultations within apps is noted as a desirable feature, as well as having the option to book appointments directly through apps. Women also want the option to sync their desktop or phone calendar with apps to remind them of medical appointments and prescription requests, as some researchers noted:

Women could see the potential usefulness of being reminded to order their next prescription through the electronic alerts system. They found managing the monthly prescription requests challenging long-term and found setting up the reminder easy with the alert popping up on their phone or tablet.

28

The application also allows women to set appointment reminders to ensure she is not missing her appointments and developing gaps in her care [...] “It allows me to remain organized for my visits to my OB with concerns, questions, symptoms I have experienced since my last visit.”

29

Issues that negatively impact user engagement and outcomes are the inability to edit information or unsubscribe from notifications, which are sometimes thought to be either inconvenient or intrusive, as well as the presence of advertisements within the app. Ease of use is essential as apps or websites that are difficult to navigate can discourage users from engaging with them. For example, a study including the use of a mobile phone app reported the following:

A hindrance and disliked aspect was the difficulty in navigating through the app (eg, no back button, clunkiness, and the inability of participants to edit their inputted daily goals) as well as a lack of color and visuals within the app, giving it a clinical appearance.

30

Some women are not comfortable answering questions that they consider intrusive, such as those related to sexual health. They rely on applications to provide trusted information about their condition and want suggestions for additional resources such as website links and local information.

##### Personalization

Women generally expressed a desire for greater personalization across several features within digital health tools. Messages and notifications that are personalized to the user’s health and self-tracking history and goals are more motivational and less likely to be ignored or perceived as irritating. Even factors such as using a first name in messages from the app make women feel like the messages are more personal and supportive.

Users have individual preferences when it comes to the frequency and timing of notifications, and it is important for digital health apps to allow for the customization of these settings as they can greatly impact user engagement and adherence. Moreover, users expressed a desire for the ability to customize their goals and the metrics they tracked. For example, they may want to change their goals in an app when their life circumstances change (eg, moving, starting a new job, becoming pregnant, or sustaining an injury). The ability to customize the dashboard of an app or website according to the user’s goals was also expressed as a desired feature. The ability to make these customizations will improve their adherence in the long run as their goals evolve.

When it comes to wearables, their placement on the body influences users’ preference and adherence to their use. For example, some women may prefer a wrist-worn device, whereas others may prefer a chest strap, a ring, or a device worn on the waist or ankle. The type of activity being monitored may also influence placement preference. A wrist-worn device may be more appropriate for monitoring steps, whereas a chest strap may be better suited for monitoring heart rate during exercise. Furthermore, placement preference may also be influenced by factors such as comfort, convenience, and visibility. A user may prefer a wrist-worn device because it is more visible and easier to access, whereas another user may prefer a device worn on the waist because it is less obtrusive and more comfortable during exercise or sleep. For instance, one study found the following:

Eight of the participants (40%) reported at some point of the long study period that the smart wristbands were uncomfortable to wear, especially at night. The wristbands irritated the skin, possibly due to pregnancy-related swelling.

31

Finally, users have different preferences for how they want information to be presented in an app or website. Some people prefer to read content that is written out with citations and links to external websites. Others enjoy learning content from videos or audio recordings. When looking at their trends and progress, some users like to look at detailed graphs showing their daily progress, whereas others prefer to look at the data occasionally and only receive high-level information. The challenges concerning personalization were articulated by several authors:

It’s a difficult one. Some women want the full picture to fully understand what they are taking. Others want a black and white sketch, but not the details. They just want to know enough. Others do not want to see the picture, they just want to get on with it without knowing too much. Catering for all is a challenge.

28

##### Safety, Privacy, and Security

Women are sometimes concerned about the physical safety of certain devices. For example, some mothers worried about their wearable wristbands scratching their babies [31]. Others worried about the effects of wearable devices on their skin, as expressed by a participant:

It’s weird because it does have a little laser thing on it, and I wonder if that’s, like, harming my skin (laughing). Like, I’ll sleep in it, and when I wake up I’ll have a red spot on my arm, it’s itchy sometime or sensitive, and I think it’s because of the laser thing, but I don’t really know.

26

Some women are concerned about the privacy and security of digital health technologies and expect appropriate safeguards to be implemented in the tools they use. However, privacy and data security are not a concern for all women:

As I said, I’m very critical about patient data in general, especially in terms of data security...If you have a free app, it really depends on what happens to the private data. As a matter of fact, usually the information is stored on the app itself, and so other apps might gain access to the data easily.

32

The survey revealed a low level of concern about issues relating to privacy or security of personal data. This suggests that privacy concerns were secondary to the benefits offered by uploading personal details into apps to provide the type of customisation they seek.

33

Researchers also shared that some users perceived there to be more privacy when using an app as compared to traditional ways of communicating:

Some participants perceived the storage of their glucose levels on the smartphone as more secure than their current registration in a booklet.

34

Women, particularly those who worked outside of the home, also commented that they appreciated the added convenience and privacy of this [text-based] communication method over phone-based communication.

34

#### Theme 3: Accuracy and Credibility

In theme 3, we identified accuracy and credibility as important factors for acceptability considerations in digital health technologies.

The accuracy of digital health can impact user trust and adoption. Digital health tools enable users to keep track of their health, symptoms, and behaviors over time without relying on memory recall, which can be inaccurate or incomplete. Many studies reported that digital tracking can lead to more accurate data collection compared to paper-based methods. For example, at-home measurements of blood pressure and other vital signs have been found to be more accurate than those taken in a hospital or clinic setting. In some cases, apps are even able to accurately predict users’ menstrual cycles and mood changes. In addition, food diaries and activity trackers are often found to be more accurate when tracked within the app compared to using traditional paper-based methods. As the following participant conveyed, digital health may also make it easier for patients to tell the truth about their habits or health concerns:

I like this principle because...I know exactly, that via tablet one would admit things you wouldn’t necessarily tell the doctor or nurse. So, for starters, you can state it in the application. Of course, a conversation shouldn’t be missed afterwards, but this might make it easier for you to overcome yourself.

32

However, accuracy can still be an issue in digital health. Different devices can produce different measurements, and some devices may miscount steps, the intensity of workouts, or the quantity and quality of sleep. For example, some women reported devices not tracking their steps while pushing a grocery cart or stroller, whereas others found that their steps were overcounted due to arm movements while they were seated. In addition, some users reported that food tracking options in apps were limited and did not include foods from their culture. Therefore, users may perceive digital health tools as not being representative of their true activity, which may lead them to discontinue the use of the devices. The following participant quote refers to a wrist-worn activity tracker:

Out paddling and we’re huffing and puffing and barely breathing and this isn’t even triggering anything. So it shows [...] that our 150 minute goal is like 60 or half of that. But we’ve actually put in the effort and then you just give up after a while. Like there’s no way I can make this.

35

Women often prefer evidence-based health information (eg, explanations of conditions and symptoms and health advice) from a trustworthy source, such as an app curated from up-to-date and evidence-based research, over general internet searches. Users reported that the information provided in some apps was incomplete or inaccurate, with gaps in content or contradictory information that diminished their trustworthiness. In such cases, users may still prefer to talk to a health professional for more trustworthy information. Some women may also find it challenging to trust information that does not disclose sources as they are unsure of its reliability. Devices that are endorsed by, cite, and link to trustworthy health sources are more appealing to users. When sharing results from a web-based survey, the authors of one study reported the following:

Some respondents were speciﬁc about from where such advice should come, stating that they wanted expert, credible and up-to-date advice while others noted that they would like to see more Australian-speciﬁc or locally-based information in apps or apps that were not linked to the manufacturers of pregnancy or baby products.

33

#### Theme 4: Audience and Adoption

Our fourth theme concerns audience and adoption, which includes two subthemes: (1) demographics and inclusivity and (2) timing and circumstances.

##### Demographics and Inclusivity

One of the challenges with digital health is to avoid one-size-fits-all interventions and to strive to tailor interventions to address the specific needs of different populations. Digital health that targets specific demographic groups or specific health conditions may increase the adoption of digital tools in those populations. That said, even when targeting people with specific health conditions as the audience, attention must be paid to the language and content in apps and websites. Some researchers noted that women did not want to participate or continue in their study because they did not want to constantly be confronted with their disease. Too much of a focus on disease and ill health can deter women from engaging with the tools, as commented on by some authors:

All but one participant preferred text content that focused on health and physical activity rather than content explicit to cancer.

36

The women emphasized that less attention should be paid to chronic disease management and medication as the only treatment option. [...] it was important to explain the implications of the result of the scan and the risk of fractures in a way that will not place the women in a sickness role unnecessarily. [...] The knowledge base of osteoporosis should focus on osteoporosis as a common condition instead of a chronic bone disease.

37

Younger women are often more familiar with and more comfortable using digital technology and, therefore, are more likely to use and adhere to a digital health protocol. Users with low technology skills want more training on how to use the digital health tools properly. Little provision is made for those for whom English is not their primary language, which can limit the accessibility and usefulness of digital health interventions. Factors such as language barriers, cultural beliefs, or lack of access to technology may lead to less adoption by some people belonging to ethnic minority groups. The relevance and usefulness of digital health may also vary based on geographic location.

Digital health tools are negatively perceived by some users if not designed to be inclusive of attributes such as body type or gender. For example, users prefer applications that use pictures or models that represent a diverse range of body sizes. Digital health technologies may not be gender inclusive and can conflate sex and gender. It is important to consider the unique health needs and experiences of individuals across the gender spectrum, as several researchers reported:

Participants commented on an exercise demonstration video and recommended that the model should have an “everyday-look” (e.g. plain clothes, jewellery). Also a choice of models of different ages to engage a wider range of patients and help them to relate or identify with the model was proposed.

38

[Participant quote]: Maybe the body image it presents...like on a lot of apps, the people doing it looked like they were athletes already. And maybe they should have more people that look normal.

39

Two women commented on the gendered design of most FTAs. FTA092 commented that “I chose Clue because it’s the only app that wasn’t pink.” FTA051 also found the gendered design of her previous app insulting; “my last app had a pink flower and was called MyDays or something ...I felt like they were trying to lure me in with this kind of ‘women’s’ approach” (FTA051). She subsequently stopped using that app and downloaded Clue.

40

##### Timing and Circumstances

Individuals are more motivated to use digital health tools during times of illness or when they have a specific health goal in mind. The introduction of technology at the appropriate time impacts the utility and effectiveness of digital health interventions, especially when they are integrated into existing health care systems and routines. Digital health apps need to account for existing medical conditions or medical history to ensure accurate and complete information. For example, technologies that do not provide an option to indicate current pregnancy are perceived as frustrating to users as the in-app goals or notifications can be irrelevant and inconsiderate of their current limitations. In a focus group, one mother shared the following:

I get frustrated with the Garmin [smartwatch] because I wear my watch during the night so it tracks my sleeping as well. Then it gives you like an insight—so a little note will pop up and you know whether your sleep has been really regular or you’ve had irregular sleep. I wish that there was a thing that during pregnancy where that I could put in and say I’m pregnant, because I got those notes that your sleep is really irregular, and I was like, “Because I’m pregnant!”

23

Users who are not experiencing symptoms or who perceive their health to be good are less likely to adopt digital health tools as they may not perceive any benefit from using them. Moreover, those who are already tracking their health using other methods (eg, paper-based tracking) are less interested in trying a new digital health tool. Similarly, regarding wearables, some people may already have a wearable and be less interested in having an additional wearable device.

#### Theme 5: Impact on Community and Health Service

This theme considers the impact of PGHD on community and health service, with three subthemes: (1) communication and community support, (2) clinical integration, and (3) health care provider perspective.

##### Communication and Community Support

One of the many perceived benefits by users of digital health interventions is the sense of community that these platforms enable. Even though some women reported feeling uncomfortable sharing personal information with strangers in a virtual group, most found that the ability to connect with others who shared similar experiences provided a sense of belonging and support that was motivating and reassuring, as shared by one woman:

What I did love about the apps is the forums. So if you have a weird pain or, you know, you have cramp in your legs at three a.m., you can get on your phone straight away, and you can get support by the women who are going through the same thing.

41

Discussion forums and social media platforms associated with digital health interventions are perceived as helpful for connecting with others, sharing personal stories, and receiving support. Digital health interventions can also help women elicit support from friends and family to stay motivated and achieve health goals. For example, researchers who reported on women’s experiences of an app for stress urinary incontinence shared that some participants found it easier to talk to friends about an app for pelvic floor muscle training rather than talk about incontinence [24]. This can enable increased accountability and further encourage adherence to the intervention. One woman spoke about how her family supported her engagement with a digital health intervention for physical activity maintenance among female cancer survivors:

My husband’s a good motivator. When I say I’m going for a walk, he’ll go with me...with my sister-in-law and her kids, it’s they want to go with me; so it’s how many steps have you got today? Or, are we going to go for a walk. That kind of thing. And with my husband and my daughter it’s, “how many steps did you get today, did you do your workout, let us get it going.”

36

In addition to support from family, friends, and community members, these digital platforms can provide an alternative to speaking with a health care provider in person. Asynchronous communication with health care providers is helpful especially for those who may not have easy access to in-person visits or for those who are uncomfortable discussing sensitive information face-to-face. Records of PGHD can also improve the ability to gather and share details with health care providers about symptoms that are difficult to remember during an in-person visit.

##### Clinical Integration

Women are more willing to participate in digital health interventions if they perceive that they have a direct impact on their clinical care. They appreciate the idea that their health is being monitored and that someone is keeping an eye on their data. Furthermore, women want to see more integration of their clinical test results within their digital health apps and websites. This increases their motivation to adhere to the interventions prescribed through the digital health application.

It was noted that physicians and other health care providers play a crucial role in promoting the use of digital health interventions among patients. As noted in the following participant quote, women enjoy being able to communicate with a health care provider through digital health:

I like it because you can tell the doctor what’s going on and submit it to your doctor, that is the main reason I like it because you can talk directly to your doctor and tell them what is going on without going in or calling.

42

Women are more likely to adopt and use technology if it is recommended by their health care providers, family members, or friends. Women reported that digital health interventions were more effective when they were supported by a health care team. For example, having access to a health coach or counselor or receiving feedback from a health care provider on their progress increases their motivation to adhere to the interventions. This support also provides reassurance that they are on the right track toward achieving their health goals. However, some patients become frustrated when they receive conflicting advice from the digital health tool and their health care provider.

##### Health Care Provider Perspective

Some articles included thoughts from health care providers on digital health tools collecting PGHD [20,37,43-55]. From the health care provider perspective, digital health can offer several benefits, including the ability to monitor patients’ adherence to treatment and interventions. This can be particularly helpful for patients with chronic conditions that require ongoing management. Providers can use digital health tools to track patients’ progress and identify any potential issues that may require further attention, which can lead to improved clinical outcomes and reduce unnecessary consultations. For instance, one provider learned about their patient’s anxiousness through a mobile health intervention:

I didn’t know my patient was feeling anxious...But when she wrote it down, we could talk about it...

43

Some health care providers expressed that digital health tracking could give them a more accurate picture of their patients’ activities and adherence to treatments. In a study about perspectives on a sensor attached to pills that can send data such as date and time of ingestion, a provider commented the following:

A positive would be data and getting a better grip on compliance. (...) I’m making sure the patient is adhering - assuming that the patient is taking everything inside of that blister, you can have confirmation of that.

44

In addition, digital health can improve the efficiency of care delivery by providing education and resources directly to patients. This can help patients better understand their condition, treatment options, and self-management strategies, which can lead to better health outcomes.

However, it was also noted that digital health interventions should not replace in-person visits but rather complement them. Some health care providers are concerned about overreliance on digital health tools as well as the potential for misinterpretation of the data they provide. There may be a lack of feedback on the correct use of interventions, such as interpretations of medical advice provided, and health care providers have raised concerns about the safety and trustworthiness of the medical advice generated by the digital health tools. Health care providers especially worry about medico-legal effects of having information from digital health tools taken out of context or without considering the full picture of the user’s history and health, as demonstrated in the following quotes:

As a health care professional, I’m just mindful that if there was a video of me up there talking, if that was taken out of context or shared with another person where that information was not appropriate, that’s a concern to me.

45

One anesthesiologist raised, “Who has access to the responses that I provide? Because if a patient receives information from me which they hold onto and is taken out of context, in a medical–legal situation, then that’s a big issue as well.”

46

Providers may also find that the abundance of information generated by digital health tools can be overwhelming and time-consuming to manage, adding to an already hectic workflow and blurring professional boundaries. Large volumes of alerts and notifications from digital health tools can be disruptive to health care providers, who expressed the need to set boundaries regarding how and when they engaged with digital health tools. In a study reporting on perspectives about digital health from key informants (health care providers and researchers), one participant shared their thoughts on the potential for digital health to increase workload and liability:

Sometimes the more information that we provide for them (doctors), the more work and liability we give them, right? So if they get so much information that becomes actionable but they are overwhelmed, now they would be obligated to do something with this patient, they are in a chain of distribution, a chain of liability.

44

#### Theme 6: Impact on Health and Behavior

Finally, our sixth theme describes the impact of PGHD on health and health behaviors.

Several studies reported that digital health interventions helped users stay motivated and, in turn, improved their health habits and behaviors, such as adherence to medication, physical activity, and healthy eating. The ability of users to look back at their data helps them identify patterns in their health and behaviors, which increases their awareness of their health and habits. The awareness then allows them to be more mindful of their habits and encourages self-reflection, thus promoting a deeper understanding of their health and well-being. The tracking of patterns in their health, combined with the educational component of some digital health tools, helps users come up with better self-management strategies and feel more confident in their ability to reach their health goals, giving them a greater sense of self-efficacy and control over their health. In a digital health intervention aimed at treating lymphedema following breast cancer treatment, a participant spoke of changes in her awareness of symptoms and improvements:

It helped me realize that I had excess fluid. My arms got lighter each time I did the exercises. My arms began to feel less heavy. It noticed it in my clothes as well.

56

Digital health interventions are often reported to positively impact the mental health and well-being of individuals. Women reported improvements in their mood, emotional state, and coping abilities. They also reported a reduction in stress and anxiety levels, which can lead to improvements in overall health outcomes. The digital health tools provide users with a sense of support and accountability as well as feelings of accomplishment when meeting their goals.

However, it is important to note that, while digital health interventions can have many benefits, they may not be suitable for everyone and may even have negative effects on some individuals. For example, some users reported increased anxiety due to excessive monitoring or notifications, and others reported negative effects on their thoughts or worsening of symptoms related to health conditions. Some users found that self-tracking made them more attached to their phones, less likely to engage in social activities, and more isolated overall. Care should be taken to ensure that users do not become obsessive about self-tracking as this can be counterproductive or even harmful. Being hyperfocused on their symptoms or health condition could be distressing and even detrimental to their overall well-being. Therefore, it is important to carefully monitor the use of digital health interventions and adjust them as needed to ensure the best possible outcomes for each individual. One woman spoke about her overreliance on an app used to track breastfeeding:

I stopped using it because um I thought I’m being too anal about this...being too concerned about it, I just need to stress less, and just go with the flow and just be a bit more relaxed about it...so, that’s why I stopped using it completely, and then I think the breastfeeding improved from there ’cause I was worrying about it less.

57

Table 2 provides a summary of the thematic analysis grouped into barriers and facilitators. It is worth noting that many things are both a barrier and a facilitator (eg, cost) depending on the individual. In addition, the presence of a specific feature may be a facilitator, whereas the absence of it may be a barrier.

**Table 2:** Summary of barriers to and facilitators of women’s use of digital health.

Theme	Barriers	Facilitators
Accessibility and connectivity	Extra costs Uncomfortable physical design (for wearables) Short battery life Not compatible with daily activities Network connectivity and device compatibility Unreliable software	Affordable Convenient
Design and functionality	Poor formatting or image quality Too noticeable or medical Inability to silence notifications and advertisements Complex navigation Intrusive questions Physical safety Trust, privacy, and security	Streamlined user interface Pleasant to look at Decision support and clinical interpretation of data Notifications or reminders and progress input Graphical data summaries Gamification Feature and display customization Personalization of content and output Trust, privacy, and security
Accuracy and credibility	Validity of measures Limited data entry options Failure to disclose information sources	Accurate sensors and algorithms Reliable data source Evidence-based advice
Audience and adoption	Too much focus on disease Low digital literacy Language and cultural barriers	Designed for a specific user group High digital literacy Inclusive language and images Focused health goals Accounts for current health state
Impact on community and health service	Overreliance on digital health Potential for misinterpretation of data Too much information output Blur professional boundaries	Creation of community Facilitates communication Social support from friends and family Asynchronous or more accessible alternative to in-person visits Supportive care providers Integration with clinical care Continuation of care Education provision
Impact on health and behavior	Stress caused by hyperfocus on tracking data Decreased engagement in social activities	Motivational tool Supports self-awareness and self-reflection through pattern recognition Increases self-efficacy Improves symptoms Reduces stress

## Discussion

### Principal Findings

In this scoping review, we summarized information from 406 articles on digital technologies collecting PGHD and how they have been used in women’s health research. We found a steady increase in articles meeting our inclusion criteria from 2015 to 2020, indicating an increasing trend in the uptake and use of digital health tools in women’s health research before the COVID-19 pandemic. Most included studies (310/406, 76.4%) were feasibility or acceptability studies, effectiveness studies, or reports of digital tool prototypes. Most studies (299/406, 73.6%) focused on tracking conditions related to pregnancy or the postpartum period, cancer survivorship, or menstrual, sexual, and reproductive health. Several types of digital health were represented, with the most common being apps, wearable devices, and websites or patient portals. Through our thematic analysis, we found several considerations of facilitators of and barriers to using digital health tools, including the accessibility and convenience of the tools, visual appearance, device functionality and ability to personalize the user experience, and accuracy of the algorithms and information provided. It is also important to consider the target audience to optimize the adoption of the tools. Engagement with digital health tools may help users improve their health and health-related behaviors and gather support from friends, family, and other digital health users. Women are more likely to use digital health if it is recommended by a health care provider, but there are both benefits and challenges that health care providers may face if considering integrating digital health technology into clinical practice.

A previously published scoping review focused on information and communications technologies as a tool for women’s empowerment [59]. They reported that the concept of empowerment appeared in various ways with no clear consensus on the definition, with some studies mentioning terms such as self-concept, self-esteem, self-worth, and self-efficacy. Our thematic analysis also found that some women’s use of digital health tools increases their self-efficacy in managing their health. Another systematic review of 13 digital health interventions for midlife women found that many interventions did not use a specific behavior change theory [60]. Our scoping review did not examine the effectiveness of the interventions described, but those designing digital health tools and interventions may want to carefully consider behavioral theories in the design to increase adoption and retention rates and adherence to interventions.

Overall, digital health technology to collect PGHD has gained popularity over the past several years. The integration of wearables, smartphones, and digital health technologies has enabled the integration of passive data collection. This wealth of data provides valuable insights into various aspects of health, enabling informed decisions and the adoption of proactive measures to improve well-being. The uptake of this technology will usher in a new era in how we manage our health and well-being. This transformation has changed how we engage with our health and shifted our perception of health and the approach we take toward maintaining it.

Femtech, as a subset of digital health technology, has grown in popularity. This was evidenced by the large increase in the number of articles published between 2015 and 2020 that used digital health tools to track metrics during pregnancy and the postpartum period as well as metrics related to menstrual, sexual, and reproductive health. These technologies empower women and people assigned female at birth to take charge of their health. This is particularly relevant for people with conditions that are not diseases or health concerns per se but are nevertheless part of managing their overall health and well-being. In this way, femtech can provide a greater sense of control over reproductive health and choices, which can be precarious in many settings worldwide. However, in a previous scoping review, researchers reported that many mobile health apps do not follow data privacy, sharing, and security standards [61]. Issues related to the privacy and security of personal health data may be especially important when it comes to tracking reproductive health in settings where sexual and reproductive health rights are not guaranteed. This focus on pregnancy and reproductive health is consistent with the fact that women’s health research has largely focused on reproductive health topics [62]. Researchers and digital health developers must address gaps in women’s health regarding areas that are not strictly related to reproductive health. Women’s health encompasses much more than obstetrics and gynecology; even for health conditions that affect men and women, there may be sex or gender differences in disease presentation, personal experiences, and treatment plans. While using gendered language and design in femtech has the potential to reinforce stereotypes regarding femininity that could cause harm [63], there is a need for apps to provide content relevant to female populations while being gender inclusive and conscious of biases in the language and advice presented.

When analyzing themes related to acceptability, personalization emerged as a key aspect influencing the adoption and sustained use of digital health tools. People respond positively and want to engage with tools that cater to their unique needs and preferences. The ability to customize elements such as the frequency of notifications, specific health measures tracked and displayed, goal-setting options, and the amount of health information provided enhanced user engagement and motivation. However, offering too many personalization options might overwhelm users, making apps or devices cumbersome to use and navigate. Simplicity and ease of use should not be compromised in the pursuit of personalization. Creating personalized experiences that are intuitive and user-friendly while integrating multiple functionalities into a given device is an important consideration. Recognizing that a “one-size-fits-all” approach is inadequate, digital intervention designers need to define their target audience clearly. Apps that cater to specific groups, such as those with certain chronic health conditions, may inherently provide a sense of personalization by addressing their unique requirements. We have also learned the importance of ensuring that the design is inclusive and accessible to everyone within the target audience. Our findings that some tools are not sensitive to certain circumstances such as pregnancy are consistent with those of a systematic review of digital health interventions for postpartum women, in which the authors reported that barriers related to postpartum status could make it more difficult to engage with the interventions [58]. Tools designed with these circumstances in mind may be more engaging for women during pregnancy and the postpartum period, leading to greater adoption and quality of the technologies. Attrition can be high among users of digital health interventions [64,65], but most participants were willing to self-track when motivated by a specific health condition.

An important finding of this review was the growing demand and expectation that PGHD are integrated with clinical care. As digital health continues evolving, patients seek more seamless interactions between digital health data and health care providers. Moreover, services delivered through digital health technologies were not expected to replace the role of health care professionals but rather to be a useful tool to support health care management. Maintaining the human touch during communication for health care delivery was seen as important, with technology complementing clinical care to enhance the overall experience for patients and providers.

One of the critical considerations in clinical integration is the accuracy of PGHD collected from digital health tools. Ensuring the reliability and validity of the data is essential for effective clinical decision-making. Striking a balance between patient empowerment and health care provider oversight is crucial to achieving the best possible outcomes. In general, it is important for health care providers to actively propose digital health during patient visits and encourage its use. While challenges and concerns associated with the use of digital health are noted from health care providers’ perspective, such as concerns about medico-legal effects, maintaining professional boundaries, and not adding an abundance of work, the benefits of these tools in supporting patient care and improving outcomes are perceived as important.

### Strengths, Limitations, and Future Directions

There are some limitations to this scoping review. Our inclusion criteria did not cover conference abstracts, conference reviews, editorials, letters, comments, or gray literature. Our review also did not include articles written in languages other than English. Therefore, there may be other uses of PGHD in women’s health that were not captured in this review. The assessments of the quality of included articles, the effectiveness of the interventions, or the accuracy in validating PGHD were outside this review’s scope and were not performed. Our aim was to provide a broad overview of PGHD in published women’s health research literature rather than evaluating the quality of the digital technologies or intervention effectiveness. Another limitation is the rapid growth of digital health and femtech, especially during the COVID-19 pandemic. It is important to note that this scoping review only captures the use of PGHD in women’s health before the emergence of the pandemic. We suggest that this review may provide a baseline for comparison in a future scoping review that captures articles published in March 2020 or later. The strengths of this review include the large number of publications analyzed and the data charting process conducted in duplicate by 2 reviewers. The broad scope of this review also helps provide an overall picture of digital health for women and highlights gaps in the research literature.

Future endeavors in this space should consider digital health tools for women for nonreproductive topics such as chronic health conditions that primarily affect women or conditions that have sex or gender differences in presentation and treatment. Within reproductive health, there was a large focus on pregnancy, but there is an unmet need for research and digital health tools appropriate for women in perimenopause and menopause. A previous literature review found <5 articles published between 2010 and 2020 about digital health technologies that meet the psychosocial needs of women experiencing menopause [66]. There may also be further opportunities for digital health tools geared toward specific racial or ethnic groups that are culturally sensitive and available in multiple languages. A systematic review found that barriers to the use of digital health among culturally and linguistically diverse populations include lower literacy levels and the use of complex medical terminology in some apps, lack of recognition of cultural concerns, stereotypes, and inaccurate portrayals of cultural groups [67]. Previous scoping reviews in the space of women’s digital health have identified the need for femtech to pay more attention to cultural appropriateness and consider cultural contexts in their design [68,69].

### Conclusions

In conclusion, the integration of wearables, smartphones, and other forms of digital health has revolutionized how we approach and engage with our health. Personalization, inclusivity, and integration with clinical care are vital aspects of developing effective digital health solutions. By understanding the needs of the target audience, providing meaningful personalization, and ensuring data accuracy, digital health can truly transform health care and empower individuals to take charge of their well-being while maintaining a collaborative relationship with health care professionals.

## Appendix

Multimedia Appendix 1 PRISMA-ScR (Preferred Reporting Items for Systematic Reviews and Meta-Analyses extension for Scoping Reviews) checklist.

Multimedia Appendix 2 Full search strategy.

Multimedia Appendix 3 List of included articles by health area.

Multimedia Appendix 4 Metrics collected in the included studies.

## Abbreviations

PGHD: person-generated health data
PRISMA: Preferred Reporting Items for Systematic Reviews and Meta-Analyses
PRISMA-ScR: Preferred Reporting Items for Systematic Reviews and Meta-Analyses extension for Scoping Reviews

## Data Availability

The data sets generated during this study are available in the OSF repository [70].

## References

[1] (2018). Conceptualizing a data infrastructure for the capture, use, and sharing of patient-generated health data in care delivery and research through 2024. The Office of the National Coordinator for Health Information Technology.

[2] DeSilva J, Prensky-Pomeranz R, Zweig M (2021). Digital health consumer adoption report 2020. RockHealth.

[3] Taylor P Penetration of mobile devices in Canada as share of the population from 2009 to 2021. Statista.

[4] Olsen E (2021). Digital health apps balloon to more than 350,000 available on the market, according to IQVIA report. MobiHealthNews.

[5] Paré G, Leaver C, Bourget C (2018). Diffusion of the digital health self-tracking movement in Canada: results of a national survey. J Med Internet Res.

[6] Fox S, Duggan M (2013). Tracking for health. Pew Research Center.

[7] Peek N, Sujan M, Scott P (2020). Digital health and care in pandemic times: impact of COVID-19. BMJ Health Care Inform.

[8] Budd J, Miller BS, Manning EM, Lampos V, Zhuang M, Edelstein M, Rees G, Emery VC, Stevens MM, Keegan N, Short MJ, Pillay D, Manley E, Cox IJ, Heymann D, Johnson AM, McKendry RA (2020). Digital technologies in the public-health response to COVID-19. Nat Med.

[9] Bruining N (2021). The post-pandemic legacy: the breakthrough of digital health and telemedicine. Cardiovasc Res.

[10] Meskó B (2022). COVID-19's impact on digital health adoption: the growing gap between a technological and a cultural transformation. JMIR Hum Factors.

[11] Kim KK, Jalil S, Ngo V, Edmunds M, Hass C, Holve E (2019). Improving self-management and care coordination with person-generated health data and mobile health. Consumer Informatics and Digital Health.

[12] Shapiro M, Johnston D, Wald J, Mon D (2012). Patient-generated health data. White paper. RTI International.

[13] Vodicka E, Kim K, Devine EB, Gnanasakthy A, Scoggins JF, Patrick DL (2015). Inclusion of patient-reported outcome measures in registered clinical trials: evidence from ClinicalTrials.gov (2007-2013). Contemp Clin Trials.

[14] (2018). Femtech—time for a digital revolution in the women’s health market. Frost & Sullivan.

[15] Karim JL, Talhouk A (2021). Person-generated health data in women's health: protocol for a scoping review. JMIR Res Protoc.

[16] Tricco AC, Lillie E, Zarin W, O'Brien KK, Colquhoun H, Levac D, Moher D, Peters MD, Horsley T, Weeks L, Hempel S, Akl EA, Chang C, McGowan J, Stewart L, Hartling L, Aldcroft A, Wilson MG, Garritty C, Lewin S, Godfrey CM, Macdonald MT, Langlois EV, Soares-Weiser K, Moriarty J, Clifford T, Tunçalp Ö, Straus SE (2018). PRISMA extension for scoping reviews (PRISMA-ScR): checklist and explanation. Ann Intern Med.

[17] Ahmadvand A, Kavanagh D, Clark M, Drennan J, Nissen L (2019). Trends and visibility of "digital health" as a keyword in articles by JMIR publications in the new millennium: bibliographic-bibliometric analysis. J Med Internet Res.

[18] Lau Y, Cheng LJ, Chi C, Tsai C, Ong KW, Ho-Lim SS, Wang W, Tan KL (2018). Development of a healthy lifestyle mobile app for overweight pregnant women: qualitative study. JMIR Mhealth Uhealth.

[19] Maxwell H, O’Shea M, Stronach M, Pearce S (2019). Empowerment through digital health trackers: an exploration of Indigenous Australian women and physical activity in leisure settings. Ann Leis Res.

[20] Hughson JP, Daly JO, Woodward-Kron R, Hajek J, Story D (2018). The rise of pregnancy apps and the implications for culturally and linguistically diverse women: narrative review. JMIR Mhealth Uhealth.

[21] Ranzenhofer LM, Engel SG, Crosby RD, Haigney M, Anderson M, McCaffery JM, Tanofsky-Kraff M (2016). Real-time assessment of heart rate variability and loss of control eating in adolescent girls: a pilot study. Int J Eat Disord.

[22] James DC, Harville C 2nd, Whitehead N, Stellefson M, Dodani S, Sears C (2016). Willingness of African American women to participate in e-Health/m-Health research. Telemed J E Health.

[23] Lupton D, Maslen S (2018). The more-than-human sensorium: sensory engagements with digital self-tracking technologies. Senses Soc.

[24] Asklund I, Samuelsson E, Hamberg K, Umefjord G, Sjöström M (2019). User experience of an app-based treatment for stress urinary incontinence: qualitative interview study. J Med Internet Res.

[25] Huberty J, Ehlers DK, Kurka J, Ainsworth B, Buman M (2015). Feasibility of three wearable sensors for 24 hour monitoring in middle-aged women. BMC Womens Health.

[26] Haney AC (2018). Young female college millennials' intent for behavior change with wearable fitness technology. Walden University.

[27] Leonard NR, Silverman M, Sherpa DP, Naegle MA, Kim H, Coffman DL, Ferdschneider M (2017). Mobile health technology using a wearable sensorband for female college students with problem drinking: an acceptability and feasibility study. JMIR Mhealth Uhealth.

[28] Brett J, Boulton M, Watson E (2018). Development of an e-health app to support women prescribed adjuvant endocrine therapy after treatment for breast cancer. Patient Prefer Adherence.

[29] Chaudhry BM (2018). Expecting great expectations when expecting. Mhealth.

[30] Mann D, Riddell L, Lim K, Byrne LK, Nowson C, Rigo M, Szymlek-Gay EA, Booth AO (2015). Mobile phone app aimed at improving iron intake and bioavailability in premenopausal women: a qualitative evaluation. JMIR Mhealth Uhealth.

[31] Grym K, Niela-Vilén H, Ekholm E, Hamari L, Azimi I, Rahmani A, Liljeberg P, Löyttyniemi E, Axelin A (2019). Feasibility of smart wristbands for continuous monitoring during pregnancy and one month after birth. BMC Pregnancy Childbirth.

[32] Goetz M, Müller M, Matthies LM, Hansen J, Doster A, Szabo A, Pauluschke-Fröhlich J, Abele H, Sohn C, Wallwiener M, Wallwiener S (2017). Perceptions of patient engagement applications during pregnancy: a qualitative assessment of the patient's perspective. JMIR Mhealth Uhealth.

[33] Lupton D, Pedersen S (2016). An Australian survey of women's use of pregnancy and parenting apps. Women Birth.

[34] de Mooij MJ, Hodny RL, O'Neil DA, Gardner MR, Beaver M, Brown AT, Barry BA, Ross LM, Jasik AJ, Nesbitt KM, Sobolewski SM, Skinner SM, Chaudhry R, Brost BC, Gostout BS, Harms RW (2018). OB nest: reimagining low-risk prenatal care. Mayo Clin Proc.

[35] Kokts-Porietis RL, Stone CR, Friedenreich CM, Froese A, McDonough M, McNeil J (2019). Breast cancer survivors' perspectives on a home-based physical activity intervention utilizing wearable technology. Support Care Cancer.

[36] Gell NM, Tursi A, Grover KW, Dittus K (2020). Female cancer survivor perspectives on remote intervention components to support physical activity maintenance. Support Care Cancer.

[37] Ravn Jakobsen P, Hermann AP, Søndergaard J, Wiil UK, Clemensen J (2018). Development of an mHealth application for women newly diagnosed with osteoporosis without preceding fractures: a participatory design approach. Int J Environ Res Public Health.

[38] Harder H, Holroyd P, Burkinshaw L, Watten P, Zammit C, Harris PR, Good A, Jenkins V (2017). A user-centred approach to developing bWell, a mobile app for arm and shoulder exercises after breast cancer treatment. J Cancer Surviv.

[39] Depper A, Howe PD (2016). Are we fit yet? English adolescent girls’ experiences of health and fitness apps. Health Sociol Rev.

[40] Gambier-Ross K, McLernon DJ, Morgan HM (2018). A mixed methods exploratory study of women's relationships with and uses of fertility tracking apps. Digit Health.

[41] Lupton D (2017). ‘It just gives me a bit of peace of mind’: Australian women’s use of digital media for pregnancy and early motherhood. Societies.

[42] Logsdon MC, Lauf A, Stikes R, Revels A, Vickers-Smith R (2020). Partnering with new mothers to develop a smart phone app to prevent maternal mortality after hospital discharge: a pilot study. J Adv Nurs.

[43] Wright AA, Raman N, Staples P, Schonholz S, Cronin A, Carlson K, Keating NL, Onnela JP (2018). The HOPE pilot study: harnessing patient-reported outcomes and biometric data to enhance cancer care. JCO Clin Cancer Inform.

[44] de Mendoza AH, Cabling ML, Dilawari A, Turner JW, Fernández N, Henderson A, Zhu Q, Gómez S, Sheppard VB (2019). Providers' perspectives on adherence to hormonal therapy in breast cancer survivors. Is there a role for the digital health feedback system?. Health Technol (Berl).

[45] Willcox JC, van der Pligt P, Ball K, Wilkinson SA, Lappas M, McCarthy EA, Campbell KJ (2015). Views of women and health professionals on mHealth lifestyle interventions in pregnancy: a qualitative investigation. JMIR Mhealth Uhealth.

[46] Ke JX, George RB, Wozney L, Chorney JL (2019). Patient-centred perioperative mobile application in Cesarean delivery: needs assessment and development. Can J Anaesth.

[47] Firet L, de Bree C, Verhoeks CM, Teunissen DA, Lagro-Janssen AL (2019). Mixed feelings: general practitioners' attitudes towards eHealth for stress urinary incontinence - a qualitative study. BMC Fam Pract.

[48] Garnweidner-Holme LM, Borgen I, Garitano I, Noll J, Lukasse M (2015). Designing and developing a mobile smartphone application for women with gestational diabetes mellitus followed-up at diabetes outpatient clinics in Norway. Healthcare (Basel).

[49] Grassl N, Nees J, Schramm K, Spratte J, Sohn C, Schott TC, Schott S (2018). A web-based survey assessing the attitudes of health care professionals in Germany toward the use of telemedicine in pregnancy monitoring: cross-sectional study. JMIR Mhealth Uhealth.

[50] Pais S, Parry D, Petrova K, Rowan J (2017). Acceptance of using an ecosystem of mobile apps for use in diabetes clinic for self-management of gestational diabetes mellitus. Stud Health Technol Inform.

[51] Ragavan MI, Ferre V, Bair-Merritt M (2020). Thrive: a novel health education mobile application for mothers who have experienced intimate partner violence. Health Promot Pract.

[52] Runkle J, Sugg M, Boase D, Galvin SL, C Coulson C (2019). Use of wearable sensors for pregnancy health and environmental monitoring: descriptive findings from the perspective of patients and providers. Digit Health.

[53] Sadigursky A (2018). Move my mood: development and evaluation of a mobile mental health self-help app using behavioral activation for women with postpartum depression. Alliant International University.

[54] Scherr CL, Feuston JL, Nixon DM, Cohen SA (2018). A two-phase approach to developing SNAP: an iPhone application to support appointment scheduling and management for women with a BRCA mutation. J Genet Couns.

[55] Tommasone G, Bazzani M, Solinas V, Serafini P (2016). Midwifery e-health: from design to validation of “mammastyle — Gravidanza Fisiologica”. Proceedings of the IEEE 18th International Conference on e-Health Networking, Applications and Services (Healthcom).

[56] Fu MR, Axelrod D, Guth AA, Wang Y, Scagliola J, Hiotis K, Rampertaap K, El-Shammaa N (2016). Usability and feasibility of health IT interventions to enhance Self-Care for Lymphedema Symptom Management in breast cancer survivors. Internet Interv.

[57] Dienelt K, Moores CJ, Miller J, Mehta K (2020). An investigation into the use of infant feeding tracker apps by breastfeeding mothers. Health Informatics J.

[58] Lim S, Tan A, Madden S, Hill B (2019). Health professionals' and postpartum women's perspectives on digital health interventions for lifestyle management in the postpartum period: a systematic review of qualitative studies. Front Endocrinol (Lausanne).

[59] Mackey A, Petrucka P (2021). Technology as the key to women's empowerment: a scoping review. BMC Womens Health.

[60] Sediva H, Cartwright T, Robertson C, Deb SK (2022). Behavior change techniques in digital health interventions for midlife women: systematic review. JMIR Mhealth Uhealth.

[61] Alfawzan N, Christen M, Spitale G, Biller-Andorno N (2022). Privacy, data sharing, and data security policies of women's mHealth apps: scoping review and content analysis. JMIR Mhealth Uhealth.

[62] Hallam L, Vassallo A, Pinho-Gomes AC, Carcel C, Woodward M (2022). Does journal content in the field of women's health represent women's burden of disease? A review of publications in 2010 and 2020. J Womens Health (Larchmt).

[63] Figueroa CA, Luo T, Aguilera A, Lyles CR (2021). The need for feminist intersectionality in digital health. Lancet Digit Health.

[64] Jabir AI, Lin X, Martinengo L, Sharp G, Theng YL, Tudor Car L (2024). Attrition in conversational agent-delivered mental health interventions: systematic review and meta-analysis. J Med Internet Res.

[65] Meyerowitz-Katz G, Ravi S, Arnolda L, Feng X, Maberly G, Astell-Burt T (2020). Rates of attrition and dropout in app-based interventions for chronic disease: systematic review and meta-analysis. J Med Internet Res.

[66] Cronin C, Hungerford C, Wilson RL (2021). Using digital health technologies to manage the psychosocial symptoms of menopause in the workplace: a narrative literature review. Issues Ment Health Nurs.

[67] Whitehead L, Talevski J, Fatehi F, Beauchamp A (2023). Barriers to and facilitators of digital health among culturally and linguistically diverse populations: qualitative systematic review. J Med Internet Res.

[68] Birati Y, Yefet E, Perlitz Y, Shehadeh N, Spitzer S (2022). Cultural and digital health literacy appropriateness of app- and web-based systems designed for pregnant women with gestational diabetes mellitus: scoping review. J Med Internet Res.

[69] Woodley SJ, Moller B, Clark AR, Bussey MD, Sangelaji B, Perry M, Kruger J (2023). Digital technologies for women's pelvic floor muscle training to manage urinary incontinence across their life course: scoping review. JMIR Mhealth Uhealth.

[70] (2024). Person-generated health data in women’s health: scoping review. OSF Home.

