# Supplementary material for: Person-Generated Health Data in Women’s Health: Scoping Review
Source: J Med Internet Res. 2024 May 16;26:e53327. doi: 10.2196/53327 (PMC11140278; doi:10.2196/53327)
Supplement: Multimedia Appendix 3 [file jmir_v26i1e53327_app3.pdf]

Multimedia Appendix 3

This appendix provides the full list of reports (N = 406) that were included in the scoping review, sorted into the following health areas of focus: (1) Pregnancy and postpartum, (2) Cancer, (3) Lifestyle, (4) Menstrual, sexual, and reproductive Health, (5) Chronic conditions, (6) Other, (7) No specific health area.

An asterisk (*) indicates that the report was placed into two of the seven categories.

Pregnancy and the Postpartum Period (n = 173)

1. Ahmed AH, Roumani AM, Szucs K, Zhang L, King D. The effect of interactive web-based monitoring on breastfeeding exclusivity, intensity, and duration in healthy, term infants after hospital discharge. *J Obstet Gynecol Neonatal Nurs*. 2016;45(2):143-154. doi:10.1016/j.jogn.2015.12.001

2. Akinseinde AS, Badejo JA, Malgwi RL. GRAVID: An indigenous m-health tool for smart and connected communities. *2016 Future Technologies Conference (FTC)*. 2016:1331-1334. doi:10.1109/FTC.2016.7821776

3. Alqudah A, McMullan P, Todd A, et al. Service evaluation of diabetes management during pregnancy in a regional maternity hospital: Potential scope for increased self-management and remote patient monitoring through mHealth solutions. *BMC Health Serv Res*. 2019;19(1):662. doi:10.1186/s12913-019-4471-9

4. Al-Shammari I, Roa L, Yorlets RR, et al. Implementation of an international standardized set of outcome indicators in pregnancy and childbirth in Kenya: Utilizing mobile technology to collect patient-reported outcomes. *PLoS One*. 2019;14(10):e0222978. doi:10.1371/journal.pone.0222978

5. Alves DS, de Moura Ferreira Gomes MC, de Araújo Novaes M. An obstetric application architecture for information, diagnosis and control of diabetes in high risk pregnancy. *Stud Health Technol Inform*. 2019;264:778-782. doi:10.3233/SHTI190329

6. Alves DS, da Silva ÉMA, Honorato MB, de Araújo Novaes M. Prototype of care application for obstetric telemonitoring of hypertensive syndromes in high risk pregnancy. *Stud Health Technol Inform*. 2019;264:1769-1770. doi:10.3233/SHTI190639

7. Alves DS, Times VC, da Silva ÉMA, Melo PSA, de Araújo Novaes M. Advances in obstetric telemonitoring: A systematic review. *Int J Med Inform*. 2020;134:104004. doi:10.1016/j.ijmedinf.2019.104004

* 8. Ancker JS, Mauer E, Kalish RB, Vest JR, Gossey JT. Early adopters of patient-generated health data upload in an electronic patient portal. *Appl Clin Inform*. 2019;10(2):254-260. doi:10.1055/s-0039-1683987

9. Anderson MM. *Patient Web Portal Use Among Women with Gestational Diabetes* [Doctoral Dissertation]. University of Wisconsin-Milwaukee; 2017.

10. Andrade J, Arsenio A, Duarte A. An integrated sensing platform for remote fetus continuous monitoring. *Proceedings of the International Conference on Biomedical Electronics and Devices (BIOSTEC 2015) - SmartMedDev*. 2015:250-259. doi:10.5220/0005318302500259

11. Aravamuthan A, Puzhakkal S, Thulamkunnuparambil Govindankutty J, Thomas S, Venkatraman V. Development of a mobile application based specific stress scale for gestational diabetes mellitus. *Int J Life Sci Pharma Res*. 2019;9(1):L58-L65. doi:10.22376/ijpbs/lpr.2019.9.1.l58-65

12. Arndt RZ. Homing in on the internet of things. *Mod Healthc*. 2018;48(39):18. https://www.proquest.com/trade-journals/homing-on-internet-things/docview/2118383548/se-2

13. Asri H, Mousannif H, Al Moatassime H. Comprehensive miscarriage dataset for an early miscarriage prediction. *Data Brief*. 2018;19:240-243. doi:10.1016/j.dib.2018.05.012

14. Azimi I, Pahikkala T, Rahmani AM, Niela-Vilén H, Axelin A, Liljeberg P. Missing data resilient decision-making for healthcare IoT through personalization: A case study on maternal health. *Future Gener Comput Syst*. 2019;96:297-308. doi:10.1016/j.future.2019.02.015

15. Bachiri M, Idri A, Fernández-Alemán JL, Toval A. Mobile personal health records for pregnancy monitoring functionalities: Analysis and potential. *Comput Methods Programs Biomed*. 2016;134:121-135. doi:10.1016/j.cmpb.2016.06.008

16. Bachiri M, Idri A, Fernández-Alemán JL, Toval A. Evaluating the privacy policies of mobile personal health records for pregnancy monitoring. *J Med Syst*. 2018;42(8):144. doi:10.1007/s10916-018-1002-x

17. Bartholomew ML, Soules K, Church K, et al. Managing diabetes in pregnancy using cell phone/internet technology. *Clin Diabetes*. 2015;33(4):169-174. doi:10.2337/diaclin.33.4.169

18. Bellevin K. Crafting a continuous engagement program. How mobile apps are changing the face of maternity care. *Health Manag Technol*. 2015;36(10):14-15.

19. Blatz M, Dowling D, Underwood PW, Bieda A, Graham G. A password-protected web site for mothers expressing milk for their preterm infants. *Adv Neonatal Care*. 2017;17(3):222-229. doi:10.1097/ANC.0000000000000365

20. Bobrova YO, Zhivolupova YA. Automatic detection of abnormal fetal states by means of a personal monitoring system. *2017 XX IEEE International Conference on Soft Computing and Measurements (SCM)*. 2017:782-784. doi:10.1109/SCM.2017.7970723

21. Bogaerts A, Ameye L, Bijlholt M, Amuli K, Heynickx D, Devlieger R. INTER-ACT: Prevention of pregnancy complications through an e-health driven interpregnancy lifestyle intervention - study protocol of a multicentre randomised controlled trial. *BMC Pregnancy Childbirth*. 2017;17(1):154. doi:10.1186/s12884-017-1336-2

22. Burke AE, Thaler KM, Geva M, Adiri Y. Feasibility and acceptability of home use of a smartphone-based urine testing application among women in prenatal care. *Am J Obstet Gynecol*. 2019;221(5):527-528. doi:10.1016/j.ajog.2019.06.015

23. Bush J, Barlow DE, Echols J, Wilkerson J, Bellevin K. Impact of a mobile health application on user engagement and pregnancy outcomes among Wyoming Medicaid members. *Telemed J E Health*. 2017;23(11):891-898. doi:10.1089/tmj.2016.0242

24. Caballero-Ruiz E, García-Sáez G, Rigla M, Villaplana M, Pons B, Hernando ME. A web-based clinical decision support system for gestational diabetes: Automatic diet prescription and detection of insulin needs. *Int J Med Inform*. 2017;102:35-49. doi:10.1016/j.ijmedinf.2017.02.014

25. Cai M, Tan KH, Ang SB. I-ACT: Integrated study on effect of Activity on ComplicaTions in pregnancy: Study protocol of a multiethnic prospective cohort study. *BMJ Open*. 2019;9(4):e025970. doi:10.1136/bmjopen-2018-025970

26. Chang CW, Ma TY, Choi MS, Hsu YY, Tsai YJ, Hou TW. Electronic personal maternity records: Both web and smartphone services. *Comput Methods Programs Biomed*. 2015;121(1):49-58. doi:10.1016/j.cmpb.2015.02.008

27. Chaudhry BM. Expecting great expectations when expecting. *Mhealth*. 2018;4:2. doi:10.21037/mhealth.2017.12.01

28. Cheung NW, Blumenthal C, Smith BJ, et al. A pilot randomised controlled trial of a text messaging intervention with customisation using linked data from wireless wearable activity monitors to improve risk factors following gestational diabetes. *Nutrients*. 2019;11(3):590. doi:10.3390/nu11030590

29. Choi J, Lee JH, Vittinghoff E, Fukuoka Y. mHealth physical activity intervention: A randomized pilot study in physically inactive pregnant women. *Matern Child Health J*. 2016;20(5):1091-1101. doi:10.1007/s10995-015-1895-7

30. Conway MR, Marshall MR, Schlaff RA, Pfeiffer KA, Pivarnik JM. Physical activity device reliability and validity during pregnancy and postpartum. *Med Sci Sports Exerc*. 2018;50(3):617-623. doi:10.1249/MSS.0000000000001469

31. Dahl AA. *Healthy Motivations for Moms-To-Be (Healthy MoM2B) Study: A Mobile Health Intervention Targeting Gestational Weight Gain among U.S. Women* [Doctoral Dissertation]. University of South Carolina; 2018.

32. Daly LM, Boyle FM, Gibbons K, Le H, Roberts J, Flenady V. Mobile applications providing guidance about decreased fetal movement: Review and content analysis. *Women Birth*. 2019;32(3):e289-e296. doi:10.1016/j.wombi.2018.07.020

33. de Mooij MJM, Hodny RL, O'Neil DA, et al. OB Nest: Reimagining low-risk prenatal care. *Mayo Clin Proc*. 2018;93(4):458-466. doi:10.1016/j.mayocp.2018.01.022

34. Deave T, Kendal S, Lingam R, et al. A study to evaluate the effectiveness of Best Beginnings' Baby Buddy phone app in England: A protocol paper. *Prim Health Care Res Dev*. 2019;20:e19. doi:10.1017/S1463423618000294

35. Demirci JR, Bogen DL. Feasibility and acceptability of a mobile app in an ecological momentary assessment of early breastfeeding. *Matern Child Nutr*. 2017;13(3):e12342. doi:10.1111/mcn.12342

36. Dienelt K, Moores CJ, Miller J, Mehta K. An investigation into the use of infant feeding tracker apps by breastfeeding mothers. *Health Informatics J*. 2020;26(3):1672-1683. doi:10.1177/1460458219888402

37. Drake AL, Unger JA, Ronen K, et al. Evaluation of mHealth strategies to optimize adherence and efficacy of Option B+ prevention of mother-to-child HIV transmission: Rationale, design and methods of a 3-armed randomized controlled trial. *Contemp Clin Trials*. 2017;57:44-50. doi:10.1016/j.cct.2017.03.007

38. Endo GK, Oluwayomi I, Alexandra V, Athavale Y, Krishnan S. Technology for continuous long-term monitoring of pregnant women for safe childbirth. *2017 IEEE Canada International Humanitarian Technology Conference (IHTC)*. 2017:6-10. doi:10.1109/IHTC.2017.8058200

39. Espinilla M, Medina J, García-Fernández ÁL, Campaña S, Londoño J. Fuzzy intelligent system for patients with preeclampsia in wearable devices. *Mob Inf Syst*. 2017;2017:7838464. doi:10.1155/2017/7838464

40. Faherty LJ, Hantsoo L, Appleby D, Sammel MD, Bennett IM, Wiebe DJ. Movement patterns in women at risk for perinatal depression: Use of a mood-monitoring mobile application in pregnancy. *J Am Med Inform Assoc*. 2017;24(4):746-753. doi:10.1093/jamia/ocx005

41. Fawsitt CG, Meaney S, Greene RA, Corcoran P. Surgical site infection after caesarean section? There is an app for that: Results from a feasibility study on costs and benefits. *Ir Med J*. 2017;110(9):635.

42. Fealy S, Chan S, Wynne O, et al. The Support for New Mums Project: A protocol for a pilot randomized controlled trial designed to test a postnatal psychoeducation smartphone application. *J Adv Nurs*. 2019;75(6):1347-1359. doi:10.1111/jan.13971

43. Foster J, Miller L, Isbell S, Shields T, Worthy N, Dunlop AL. mHealth to promote pregnancy and interconception health among African-American women at risk for adverse birth outcomes: A pilot study. 2015;1:20. doi:10.3978/j.issn.2306-9740.2015.12.01

44. Ganapathy R, Grewal A, Castleman JS. Remote monitoring of blood pressure to reduce the risk of preeclampsia related complications with an innovative use of mobile technology. *Pregnancy Hypertens*. 2016;6(4):263-265. doi:10.1016/j.preghy.2016.04.005

45. Garnweidner-Holme LM, Borgen I, Garitano I, Noll J, Lukasse M. Designing and developing a mobile smartphone application for women with gestational diabetes mellitus followed-up at diabetes outpatient clinics in Norway. *Healthcare (Basel)*. 2015;3(2):310-323. doi:10.3390/healthcare3020310

46. Gatti M. Feasibility of FreeStyle Libre Flash Glucose Monitoring System in pregnant woman affected by type 1 diabetes. *Acta Diabetol*. 2019;56(4):481-483. doi:10.1007/s00592-018-1252-6

47. Gianfrancesco C, Darwin Z, McGowan L, et al. Exploring the feasibility of use of an online dietary assessment tool (myfood24) in women with gestational diabetes. *Nutrients*. 2018;10(9):1147. doi:10.3390/nu10091147

48. Gill R, Ogilvie G, Norman WV, Fitzsimmons B, Maher C, Renner R. Feasibility and acceptability of a mobile technology intervention to support postabortion care in British Columbia: Phase I. *J Med Internet Res*. 2019;21(5):e13387. doi:10.2196/13387

49. Goetz M, Müller M, Matthies LM, et al. Perceptions of patient engagement applications during pregnancy: A qualitative assessment of the patient's perspective. *JMIR Mhealth Uhealth*. 2017;5(5):e73. doi:10.2196/mhealth.7040

50. Gogoi A, Katoch M, Agrawal P. Empowering women in India to influence maternal healthcare quality through mobile phones and crowdsourcing. In: *Global Perspectives on Women's Sexual and Reproductive Health Across the Lifecourse*. Springer; 2018:111-123. doi:10.1007/978-3-319-60417-6_7

51. Graham ML, Strawderman MS, Demment M, Olson CM. Does usage of an eHealth intervention reduce the risk of excessive gestational weight gain? Secondary analysis from a randomized controlled trial. *J Med Internet Res*. 2017;19(1):e6. doi:10.2196/jmir.6644

52. Grassl N, Nees J, Schramm K, et al. A web-based survey assessing the attitudes of health care professionals in Germany toward the use of telemedicine in pregnancy monitoring: Cross-sectional study. *JMIR Mhealth Uhealth*. 2018;6(8):e10063. doi:10.2196/10063

53. Grym K, Niela-Vilén H, Ekholm E, et al. Feasibility of smart wristbands for continuous monitoring during pregnancy and one month after birth. *BMC Pregnancy Childbirth*. 2019;19(1):34. doi:10.1186/s12884-019-2187-9

54. Guendelman S, Broderick A, Mlo H, Gemmill A, Lindeman D. Listening to communities: Mixed-method study of the engagement of disadvantaged mothers and pregnant women with digital health technologies. *J Med Internet Res*. 2017;19(7):e240. doi:10.2196/jmir.7736

55. Guo H, Zhang Y, Li P, Zhou P, Chen LM, Li SY. Evaluating the effects of mobile health intervention on weight management, glycemic control and pregnancy outcomes in patients with gestational diabetes mellitus. *J Endocrinol Invest*. 2019;42(6):709-714. doi:10.1007/s40618-018-0975-0

56. Gyselaers W, Lanssens D, Perry H, Khalil A. Mobile health applications for prenatal assessment and monitoring. *Curr Pharm Des*. 2019;25(5):615-623. doi:10.2174/1381612825666190320140659

57. Hadar E, Chen R, Toledano Y, Tenenbaum-Gavish K, Atzmon Y, Hod M. Noninvasive, continuous, real-time glucose measurements compared to reference laboratory venous plasma glucose values. *J Matern Fetal Neonatal Med*. 2019;32(20):3393-3400. doi:10.1080/14767058.2018.1463987

58. Halili L, Liu R, Hutchinson KA, Semeniuk K, Redman LM, Adamo KB. Development and pilot evaluation of a pregnancy-specific mobile health tool: A qualitative investigation of SmartMoms Canada. *BMC Med Inform Decis Mak*. 2018;18(1):95. doi:10.1186/s12911-018-0705-8

59. Hantsoo L, Criniti S, Khan A, et al. A mobile application for monitoring and management of depressed mood in a vulnerable pregnant population. *Psychiatr Serv*. 2018;69(1):104-107. doi:10.1176/appi.ps.201600582

60. Happillon T, Muszynski C, Zhang F, Marque C, Istrate D. Detection of movement artefacts and contraction bursts using accelerometer and electrohysterograms for home monitoring of pregnancy. *IRBM*. 2018;39(6):379-385. doi:10.1016/j.irbm.2018.10.008

61. Hawkins M, Iradukunda F, Paterno M. Feasibility of a sleep self-management intervention in pregnancy using a personalized health monitoring device: Protocol for a pilot randomized controlled trial. *JMIR Res Protoc*. 2019;8(5):e12455. doi:10.2196/12455

62. Heminger CL, Schindler-Ruwisch JM, Abroms LC. Smoking cessation support for pregnant women: Role of mobile technology. *Subst Abuse Rehabil*. 2016;7:15-26. doi:10.2147/sar.s84239

63. Hirshberg A, Downes K, Srinivas S. Comparing standard office-based follow-up with text-based remote monitoring in the management of postpartum hypertension: A randomised clinical trial. *BMJ Qual Saf*. 2018;27(11):871-877. doi:10.1136/bmjqs-2018-007837

64. Hoştină A, Cherecheş RM. The use of mHealth technology in smoking cessation interventions for pregnant women: The emerging field of eHealth and mHealth in Romania. *Transylvanian International Conference In Public Administration*. 2017:220-227. https://www.apubb.ro/intconf/wp-content/uploads/2020/07/TICPA_Proceedings_2017.pdf

65. Hughson JP, Daly JO, Woodward-Kron R, Hajek J, Story D. The rise of pregnancy apps and the implications for culturally and linguistically diverse women: Narrative review. *JMIR Mhealth Uhealth*. 2018;6(11):e189. doi:10.2196/mhealth.9119

66. Husain AM, Hassan T. Localizing pregnant women and newborns in rural areas and bridging health care gap. *2016 19th International Conference on Computer and Information Technology (ICCIT)*. 2016:546-549. doi:10.1109/ICCITECHN.2016.7860257

67. Idri A, Bachiri M, Fernández-Alemán JL. A framework for evaluating the software product quality of pregnancy monitoring mobile personal health records. *J Med Syst*. 2016;40(3):50. doi:10.1007/s10916-015-0415-z

68. Jefferson UT, Zachary I, Majee W. Employing a user-centered design to engage mothers in the development of a mHealth breastfeeding application. *Comput Inform Nurs*. 2019;37(10):522-531. doi:10.1097/CIN.0000000000000549

69. Jiménez-Serrano S, Tortajada S, García-Gómez JM. A mobile health application to predict postpartum depression based on machine learning. *Telemed J E Health*. 2015;21(7):567-574. doi:10.1089/tmj.2014.0113

70. Jones M, Smith M, Lewis S, Parrott S, Coleman T. A dynamic, modifiable model for estimating cost-effectiveness of smoking cessation interventions in pregnancy: Application to an RCT of self-help delivered by text message. *Addiction*. 2019;114(2):353-365. doi:10.1111/add.14476

71. Kapaya H, Pipkin FB, Hayes-Gill B, Loughna PV. Circadian changes and sex-related differences in fetal heart rate parameters. *Matern Health Neonatol Perinatol*. 2016;2(1):9. doi:10.1186/s40748-016-0037-6

72. Kapaya H, Dimelow ER, Anumba D. Women's experience of wearing a portable fetal-electrocardiogram device to monitor small-for-gestational age fetus in their home environment. *Women's Health (Lond)*. 2018;14:1745506518785620. doi:10.1177/1745506518785620

73. Karagiannaki K, Chonianakis S, Patelarou E, Panousopoulou A, Papadopouli M. mMamee: A mHealth platform for monitoring and assessing maternal environmental exposure. *2015 IEEE 28th Symposium on Computer-Based Medical Systems*. 2015:163-168. doi:10.1109/CBMS.2015.56

74. Ke JXC, George RB, Wozney L, Chorney JL. Patient-centred perioperative mobile application in Cesarean delivery: Needs assessment and development. *Can J Anaesth*. 2019;66(10):1194-1201. doi:10.1007/s12630-019-01392-x

75. Knight-Agarwal C, Davis DL, Williams L, Davey R, Cox R, Clarke A. Development and pilot testing of the Eating4Two mobile phone app to monitor gestational weight gain. *JMIR Mhealth Uhealth*. 2015;3(2):e44. doi:10.2196/mhealth.4071

76. Kominiarek MA, Vyhmeister H, Balmert LC, et al. Activity tracking devices in group prenatal care: A feasibility study. *Biores Open Access*. 2018;7(1):165-176. doi:10.1089/biores.2018.0021

77. Kominiarek MA, Balmert LC, Tolo H, Grobman W, Simon M. A feasibility study of activity tracking devices in pregnancy. *BMC Pregnancy Childbirth*. 2019;19(1):401. doi:10.1186/s12884-019-2557-3

78. Kumar S, Gupta Y, Mago V. Health-monitoring of pregnant women: Design requirements, and proposed reference architecture. *2019 16th IEEE Annual Consumer Communications and Networking Conference (CCNC)*. 2019:1-6. doi:10.1109/CCNC.2019.8651768

79. Kumaresh S, Sabareesh M, Srihari R. Non-invasive fetus heart rate and growth measurement with abnormality detection using IoT. *2016 International Conference on Electrical, Electronics, and Optimization Techniques (ICEEOT)*. 2016:3655-3659. doi:10.1109/ICEEOT.2016.7755390

80. Lai J, Woodward R, Alexandrov Y, et al. Performance of a wearable acoustic system for fetal movement discrimination. *PLoS One*. 2018;13(5):e0195728. doi:10.1371/journal.pone.0195728

81. Lanssens D, Vandenberk T, Smeets CJ, et al. Remote monitoring of hypertension diseases in pregnancy: A pilot study. *JMIR Mhealth Uhealth*. 2017;5(3):e25. doi:10.2196/mhealth.6552

82. Lau Y, Cheng LJ, Chi C, et al. Development of a healthy lifestyle mobile app for overweight pregnant women: Qualitative study. *JMIR Mhealth Uhealth*. 2018;6(4):e91. doi:10.2196/mhealth.9718

83. Ledford CJW, Canzona MR, Cafferty LA, Hodge JA. Mobile application as a prenatal education and engagement tool: A randomized controlled pilot. *Patient Educ Couns*. 2016;99(4):578-582. doi:10.1016/j.pec.2015.11.006

84. Ledford CJW, Womack JJ, Rider HA, et al. Unexpected effects of a system-distributed mobile application in maternity care: A randomized controlled trial. *Health Educ Behav*. 2018;45(3):323-330. doi:10.1177/1090198117732110

85. Lemelin A, Paré G, Bernard S, Godbout A. Demonstrated cost-effectiveness of a telehomecare program for gestational diabetes mellitus management. *Diabetes Technol Ther*. 2020;22(3):195-202. doi:10.1089/dia.2019.0259

86. Lenz B, Eichler A, Schwenke E, et al. Mindfulness-based stress reduction in pregnancy: An app-based programme to improve the health of mothers and children (MINDFUL/PMI Study). *Geburtshilfe Frauenheilkd*. 2018;78(12):1283-1291. doi:10.1055/a-0677-2630

87. Lim K, Chi C, Chan SY, et al. Smart Phone APP to Restore Optimal Weight (SPAROW): Protocol for a randomised controlled trial for women with recent gestational diabetes. *BMC Public Health*. 2019;19(1):1287. doi:10.1186/s12889-019-7691-3

88. Lim S, Tan A, Madden S, Hill B. Health professionals' and postpartum women's perspectives on digital health interventions for lifestyle management in the postpartum period: A systematic review of qualitative studies. *Front Endocrinol (Lausanne)*. 2019;10:767. doi:10.3389/fendo.2019.00767

89. Logsdon MC, Lauf A, Stikes R, Revels A, Vickers-Smith R. Partnering with new mothers to develop a smart phone app to prevent maternal mortality after hospital discharge: A pilot study. *J Adv Nurs*. 2020;76(1):324-327. doi:10.1111/jan.14219

90. Lupton D, Pedersen S. An Australian survey of women's use of pregnancy and parenting apps. *Women Birth*. 2016;29(4):368-375. doi:10.1016/j.wombi.2016.01.008

91. Lupton D. 'It just gives me a bit of peace of mind': Australian women's use of digital media for pregnancy and early motherhood. *Societies*. 2017;7(3):25. doi:10.3390/soc7030025

92. Mackillop LH, Bartlett K, Birks J, et al. Trial protocol to compare the efficacy of a smartphone-based blood glucose management system with standard clinic care in the gestational diabetic population. *BMJ Open*. 2016;6(3):e009702. doi:10.1136/bmjopen-2015-009702

93. Mackillop L, Hirst JE, Bartlett KJ, et al. Comparing the efficacy of a mobile phone-based blood glucose management system with standard clinic care in women with gestational diabetes: Randomized controlled trial. *JMIR Mhealth Uhealth*. 2018;6(3):e71. doi:10.2196/mhealth.9512

* 94. Mallya R, Kothari S. Organizational structure for woman's health monitoring system using OMAS. *2017 Conference on Information and Communication Technology (CICT)*. 2017:1-6. doi:10.1109/INFOCOMTECH.2017.8340587

95. Marcano Belisario JS, Doherty K, O'Donoghue J, et al. A bespoke mobile application for the longitudinal assessment of depression and mood during pregnancy: Protocol of a feasibility study. *BMJ Open*. 2017;7(5):e014469. doi:10.1136/bmjopen-2016-014469

96. Marko KI, Krapf JM, Meltzer AC, et al. Testing the feasibility of remote patient monitoring in prenatal care using a mobile app and connected devices: A prospective observational trial. *JMIR Res Protoc*. 2016;5(4):e200. doi:10.2196/resprot.6167

97. Marko KI, Ganju N, Krapf JM, et al. A mobile prenatal care app to reduce in-person visits: Prospective controlled trial. *JMIR Mhealth Uhealth*. 2019;7(5):e10520. doi:10.2196/10520

98. McLean A, Osgood N, Newstead-Angel J, et al. Building research capacity: Results of a feasibility study using a novel mHealth epidemiological data collection system within a gestational diabetes population. *Stud Health Technol Inform*. 2017;234:228-232. doi:10.3233/978-1-61499-742-9-228

99. McMillan B, Easton K, Goyder E, et al. Reducing risk of type 2 diabetes after gestational diabetes: A qualitative study to explore the potential of technology in primary care. *Br J Gen Pract*. 2018;68(669):e260-e267. doi:10.3399/bjgp18X695297

100. Mendez DD, Sanders SA, Karimi HA, et al. Understanding pregnancy and postpartum health using ecological momentary assessment and mobile technology: Protocol for the postpartum mothers mobile study. *JMIR Res Protoc*. 2019;8(6):e13569. doi:10.2196/13569

101. Mertens L, Braeken MAKA, Bogaerts A. Effect of lifestyle coaching including telemonitoring and telecoaching on gestational weight gain and postnatal weight loss: A systematic review. *Telemed J E Health*. 2019;25(10):889-901. doi:10.1089/tmj.2018.0139

102. Moreira MWL, Rodrigues JJPC, Oliveira AMB, Saleem K. Smart mobile system for pregnancy care using body sensors. *2016 International Conference on Selected Topics in Mobile and Wireless Networking (MoWNeT)*. 2016:1-4. doi:10.1109/MoWNet.2016.7496609

103. Musyoka FM, Thiga MM, Muketha GM. A 24-hour ambulatory blood pressure monitoring system for preeclampsia management in antenatal care. *Inform Med Unlocked*. 2019;16:100199. doi:10.1016/j.imu.2019.100199

104. Naughton F, Cooper S, Foster K, et al. Large multi-centre pilot randomized controlled trial testing a low-cost, tailored, self-help smoking cessation text message intervention for pregnant smokers (MiQuit). *Addiction*. 2017;112(7):1238-1249. doi:10.1111/add.13802

105. Nicholson WK, Beckham AJ, Hatley K, et al. The Gestational Diabetes Management System (GooDMomS): Development, feasibility and lessons learned from a patient-informed, web-based pregnancy and postpartum lifestyle intervention. *BMC Pregnancy Childbirth*. 2016;16(1):277. doi:10.1186/s12884-016-1064-z

106. Nikolopoulos M, Karampela I, Antonakos G, et al. Mobile phone applications for gestational diabetes mellitus: Appraisal and perspectives. *Stud Health Technol Inform*. 2019;262:39-42. doi:10.3233/SHTI190011

107. Niţulescu A, Crişan-Vida M, Stoicu-Tivadar L, Bernad E. integrated wireless sensor network for monitoring pregnant women. *Stud Health Technol Inform*. 2015;210:354-358. doi:10.3233/978-1-61499-512-8-354

108. Olson CM, Strawderman MS, Graham ML. Association between consistent weight gain tracking and gestational weight gain: Secondary analysis of a randomized trial. *Obesity (Silver Spring)*. 2017;25(7):1217-1227. doi:10.1002/oby.21873

109. Olson CM, Groth SW, Graham ML, Reschke JE, Strawderman MS, Fernandez ID. The effectiveness of an online intervention in preventing excessive gestational weight gain: The e-moms roc randomized controlled trial. *BMC Pregnancy Childbirth*. 2018;18(1):148. doi:10.1186/s12884-018-1767-4

110. Oviedo-Caro MÁ, Bueno-Antequera J, Munguía-Izquierdo D. Transcultural adaptation and psychometric properties of Spanish version of Pregnancy Physical Activity Questionnaire: the PregnActive project. *Gac Sanit*. 2019;33(4):369-376. doi:10.1016/j.gaceta.2017.12.004

111. Pais S, Parry D, Petrova K, Rowan J. Acceptance of using an ecosystem of mobile apps for use in diabetes clinic for self-management of gestational diabetes mellitus. *Stud Health Technol Inform*. 2017;245:188-192. doi:10.3233/978-1-61499-830-3-188

112. Peng J, Huang Y, Yu K, Fan R, Zhou J. Maternal health care wearing equipment based on fetal information monitoring. *J Infect Public Health*. 2020;13(12):2009-2013. doi:10.1016/j.jiph.2019.07.031

113. Perry H, Sheehan E, Thilaganathan B, Khalil A. Home blood-pressure monitoring in a hypertensive pregnant population. *Ultrasound Obstet Gynecol*. 2018;51(4):524-530. doi:10.1002/uog.19023

114. Phillips TK, Bonnet K, Myer L, et al. Acceptability of interventions to improve engagement in HIV care among pregnant and postpartum women at two urban clinics in South Africa. *Matern Child Health J*. 2019;23(9):1260-1270. doi:10.1007/s10995-019-02766-9

115. Poudyal A, van Heerden A, Hagaman A, et al. Wearable digital sensors to identify risks of postpartum depression and personalize psychological treatment for adolescent mothers: Protocol for a mixed methods exploratory study in rural Nepal. *JMIR Res Protoc*. 2019;8(8):e14734. doi:10.2196/14724.

116. Power JM, Phelan S, Hatley K, et al. Engagement and weight loss in a web and mobile program for low-income postpartum women: Fit Moms/Mamás Activas. *Health Educ Behav*. 2019;46(2_suppl):114-123. doi:10.1177/1090198119873915

117. Pustozerov EA, Chernykh VY, Popova PV., Vasyukova EA, Tkachuk AS, Yuldashev ZM. Health monitoring system for patients with gestational diabetes mellitus based on nutrition diaries and fitness bracelets. *Biomed Eng*. 2020;53(5):305-308. doi:10.1007/s10527-020-09931-3

118. Pustozerov E, Tkachuk A, Vasukova E, et al. The role of glycemic index and glycemic load in the development of real-time postprandial glycemic response prediction models for patients with gestational diabetes. *Nutrients*. 2020;12(2):302. doi:10.3390/nu12020302

119. Qing L, Weiying S. A Chinese survey of women's use and expectation of pregnancy applications. *Stud Health Technol Inform*. 2019;264:749-752. doi:10.3233/SHTI190323

120. Radin JM, Steinhubl SR, Su AI, et al. The Healthy Pregnancy Research Program: Transforming pregnancy research through a ResearchKit app. *NPJ Digit Med*. 2018;1:45. doi:10.1038/s41746-018-0052-2

121. Redman LM, Gilmore LA, Breaux J, et al. Effectiveness of SmartMoms, a novel eHealth intervention for management of gestational weight gain: Randomized controlled pilot trial. *JMIR Mhealth Uhealth*. 2017;5(9):e133. doi:10.2196/mhealth.8228

122. Regan AK, Tracey LE, Blyth CC, Richmond PC, Effler PV. A prospective cohort study assessing the reactogenicity of pertussis and influenza vaccines administered during pregnancy. *Vaccine*. 2016;34(20):2299-2304. doi:10.1016/j.vaccine.2016.03.084

123. Rhoads SJ, Serrano CI, Lynch CE, et al. Exploring implementation of m-Health monitoring in postpartum women with hypertension. *Telemed J E Health*. 2017;23(10):833-841. doi:10.1089/tmj.2016.0272

124. Rigla M, Martínez-Sarriegui I, García-Sáez G, Pons B, Hernando ME. Gestational diabetes management using smart mobile telemedicine. *J Diabetes Sci Technol*. 2018;12(2):260-264. doi:10.1177/1932296817704442

125. Rivera-Romero O, Olmo A, Muñoz R, Stiefel P, Miranda ML, Beltrán LM. Mobile health solutions for hypertensive disorders in pregnancy: Scoping literature review. *JMIR Mhealth Uhealth*. 2018;6(5):e130. doi:10.2196/mhealth.9671

126. Robu A, Gauca B, Crisan-Vida M, Stoicu-Tivadar L. Integrated system for monitoring and prevention in obstetrics-gynaecology. *Stud Health Technol Inform*. 2016;221:8-12. doi:10.3233/978-1-61499-633-0-8

127. Runkle J, Sugg M, Boase D, Galvin SL, C Coulson C. Use of wearable sensors for pregnancy health and environmental monitoring: Descriptive findings from the perspective of patients and providers. *Digit Health*. 2019;5:2055207619828220. doi:10.1177/2055207619828220

128. Sadigursky A. *Move My Mood: Development and Evaluation of a Mobile Mental Health Self-Help App Using Behavioral Activation for Women with Postpartum Depression* [Doctoral Dissertation]. Alliant International University; 2018.

* 129. Santur Y, Santur SG, Karaköse M. Architecture and implementation of a smart-pregnancy monitoring system using web-based application. *Expert Syst*. 2020;37(1):e12379. doi:10.1111/exsy.12379

130. Schramm K, Grassl N, Nees J, et al. Women's attitudes toward self-monitoring of their pregnancy using noninvasive electronic devices: Cross-sectional multicenter study. *JMIR Mhealth Uhealth*. 2019;7(1):e11458. doi:10.2196/11458

131. Sheehan E, Khalil A, Kay L. Using a smartphone app to identify signs of pre-eclampsia and/or worsening blood pressure. *Br J Midwifery*. 2019;27(2):92-99. doi:10.12968/bjom.2019.27.2.92

132. Sidhu S, Ma K, Sadovnikova A. Features and educational content related to milk production in breastfeeding apps: Content analysis informed by social cognitive theory. *JMIR Pediatr Parent*. 2019;2(1):e12364. doi:10.2196/12364

133. Skar JB, Garnweidner-Holme LM, Lukasse M, Terragni L. Women's experiences with using a smartphone app (the Pregnant+ app) to manage gestational diabetes mellitus in a randomised controlled trial. *Midwifery*. 2018;58:102-108. doi:10.1016/j.midw.2017.12.021

134. Soffer MD, Chen KT. In search of accurate fetal heart rate monitoring mobile applications. *Telemed J E Health*. 2019;25(9):870-877. doi:10.1089/tmj.2018.0104

135. Sommer J, Daus M, Simon M, Luna D. Health portals for specific populations: A design for pregnant women. *Stud Health Technol Inform*. 2018;250:3-6. doi:10.3233/978-1-61499-872-3-3

136. Souza RT, Cecatti JG, Mayrink J, et al. Identification of earlier predictors of pregnancy complications through wearable technologies in a Brazilian multicentre cohort: Maternal Actigraphy Exploratory Study I (MAES-I) study protocol. *BMJ Open*. 2019;9(4):e023101. doi:10.1136/bmjopen-2018-023101

137. Stockwell MS, Cano M, Jakob K, et al. Feasibility of text message influenza vaccine safety monitoring during pregnancy. *Am J Prev Med*. 2017;53(3):282-289. doi:10.1016/j.amepre.2017.03.014

138. Sugawara J, Ochi D, Yamashita R, et al. Maternity Log study: A longitudinal lifelog monitoring and multiomics analysis for the early prediction of complicated pregnancy. *BMJ Open*. 2019;9(2):e025939. doi:10.1136/bmjopen-2018-025939

139. Sukumar N, Dallosso H, Saravanan P, et al. Baby Steps - a structured group education programme with accompanying mobile web application designed to promote physical activity in women with a history of gestational diabetes: Study protocol for a randomised controlled trial. *Trials*. 2018;19(1):682. doi:10.1186/s13063-018-3067-8

140. Supriyanti R, Erfayanto U, Ramadani Y, Murdyantoro E, Widodo HB. Blood pressure mobile monitoring for pregnant woman based Android system. *IOP Conference Series: Materials Science and Engineering*. 2016;105:012048. doi:10.1088/1757-899X/105/1/012048

141. Symons Downs D, Savage JS, Rivera DE, et al. Individually tailored, adaptive intervention to manage gestational weight gain: Protocol for a randomized controlled trial in women with overweight and obesity. *JMIR Res Protoc*. 2018;7(6):e150. doi:10.2196/resprot.9220

142. Tang H, Wang T, Li M, Yang X. The design and implementation of cardiotocography signals classification algorithm based on neural network. *Comput Math Methods Med*. 2018;2018:8568617. doi:10.1155/2018/8568617

143. Tassone C, Keshavjee K, Paglialonga A, Moreira N, Pinto J, Quintana Y. Evaluation of mobile apps for treatment of patients at risk of developing gestational diabetes. *Health Informatics J*. 2020;26(3):1983-1994. doi:10.1177/1460458219896639

144. Thomas GM, Lupton D. Threats and thrills: Pregnancy apps, risk and consumption. *Health Risk Soc*. 2015;17(7-8):495-509. doi:10.1080/13698575.2015.1127333

145. Thornham H. Algorithmic vulnerabilities and the datalogical: Early motherhood and tracking-as-care regimes. *Convergence*. 2019;25(2):171-185. doi:10.1177/1354856519835772

146. Tommasone G, Bazzani M, Solinas V, Serafini P. Midwifery E-Health: From design to validation of "Mammastyle — Gravidanza Fisiologica." *2016 IEEE 18th International Conference on e-Health Networking, Applications and Services (Healthcom)*. 2016:1-6. doi:10.1109/HealthCom.2016.7749499

147. Tumpa SN, Islam AB, Ankon MTM. Smart care: An intelligent assistant for pregnant mothers. *2017 4th International Conference on Advances in Electrical Engineering (ICAEE)*. 2017:754-759. doi:10.1109/ICAEE.2017.8255455

148. van den Heuvel JFM, Groenhof TK, Veerbeek JHW, et al. eHealth as the next-generation perinatal care: An overview of the literature. *J Med Internet Res*. 2018;20(6):e202. doi:10.2196/jmir.9262

149. van den Heuvel JFM, Lely AT, Franx A, Bekker MN. Validation of the iHealth Track and Omron HEM-9210T automated blood pressure devices for use in pregnancy. *Pregnancy Hypertens*. 2019;15:37-41. doi:10.1016/j.preghy.2018.10.008

150. van den Heuvel JFM, Ganzevoort W, De Haan-Jebbink JM, et al. HOspital care versus TELemonitoring in high-risk pregnancy (HOTEL): Study protocol for a multicentre non-inferiority randomised controlled trial. *BMJ Open*. 2019;9(10):e031700. doi:10.1136/bmjopen-2019-031700

151. van den Heuvel JFM, Kariman SS, van Solinge WW, Franx A, Lely AT, Bekker MN. SAFE@HOME – Feasibility study of a telemonitoring platform combining blood pressure and preeclampsia symptoms in pregnancy care. *Eur J Obstet Gynecol Reprod Biol*. 2019;240:226-231. doi:10.1016/j.ejogrb.2019.07.012

152. van der Pligt P, Ball K, Hesketh KD, et al. A pilot intervention to reduce postpartum weight retention and central adiposity in first-time mothers: Results from the mums OnLiNE (Online, Lifestyle, Nutrition & Exercise) study. *J Hum Nutr Diet*. 2018;31(3):314-328. doi:10.1111/jhn.12521

153. van der Pligt P, Ball K, Hesketh KD, Crawford D, Teychenne M, Campbell K. The views of first time mothers completing an intervention to reduce postpartum weight retention: A qualitative evaluation of the mums OnLiNE study. *Midwifery*. 2018;56:23-28. doi:10.1016/j.midw.2017.09.013

154. Van Horn L, Peaceman A, Kwasny M, et al. Dietary approaches to stop hypertension diet and activity to limit gestational weight: Maternal offspring metabolics family intervention trial, a technology enhanced randomized trial. *Am J Prev Med*. 2018;55(5):603-614. doi:10.1016/j.amepre.2018.06.015

155. Vandenberk T, Storms V, Lanssens D, et al. Vendor-independent mobile health monitoring platform for digital health studies: Development and usability study. *JMIR Mhealth Uhealth*. 2019;7(10):e12586. doi:10.2196/12586

156. Wang CJ, Chaovalit P, Pongnumkul S. A breastfeed-promoting mobile app intervention: Usability and usefulness study. *JMIR Mhealth Uhealth*. 2018;6(1):e27. doi:10.2196/mhealth.8337

157. Wang N, Deng Z, Wen LM, Ding Y, He G. Understanding the use of smartphone apps for health information among pregnant Chinese women: Mixed methods study. *JMIR Mhealth Uhealth*. 2019;7(6):e12631. doi:10.2196/12631

158. Washio Y, Frederick J, Archibald A, Bertram N, Crowe JA. Community-initiated pilot program "My Baby's Breath" to reduce prenatal alcohol use. *Del Med J*. 2017;89(2):46-51.

159. Wernimont SA, Sheng JS, Fleener D, Summers KM, Syrop C, Andrews JI. Cellular-enabled glucometers and maternal glucose control: A quality improvement initiative. *J Diabetes Sci Technol*. 2020;14(1):77-82. doi:10.1177/1932296819856360

160. Willcox JC, Campbell KJ, McCarthy EA, et al. Testing the feasibility of a mobile technology intervention promoting healthy gestational weight gain in pregnant women (txt4two) - study protocol for a randomised controlled trial. *Trials*. 2015;16:209. doi:10.1186/s13063-015-0730-1

161. Willcox JC, van der Pligt P, Ball K, et al. Views of women and health professionals on mHealth lifestyle interventions in pregnancy: A qualitative investigation. *JMIR Mhealth Uhealth*. 2015;3(4):e99. doi:10.2196/mhealth.4869

162. Wiweko B, Riyanti A, Olivia S, et al. "Jakarta Reproduksi Sehat" (JAKPROS) mobile application for healthy Jakarta. *AIP Conference Proceedings*. 2019;2092(1):040005. doi:10.1063/1.5096738

163. Womack JJ, Anderson LN, Ledford CJW. Presence of complex and potentially conflicting information in prenatal mobile apps. *Health Promot Pract*. 2020;21(2):238-245. doi:10.1177/1524839918796216

164 Xydopoulos G, Perry H, Sheehan E, Thilaganathan B, Fordham R, Khalil A. Home blood-pressure monitoring in a hypertensive pregnant population: Cost-minimization study. *Ultrasound Obstet Gynecol*. 2019;53(4):496-502. doi:10.1002/uog.19041

165. Yang P, Lo W, He ZL, Xiao XM. Medical nutrition treatment of women with gestational diabetes mellitus by a telemedicine system based on smartphones. *J Obstet Gynaecol Res*. 2018;44(7):1228-1234. doi:10.1111/jog.13669

166. Yuan L, Yuan Y, Zhou Z, Bai Y, Wu S. A fetal ECG monitoring system based on the Android smartphone. *Sensors (Basel)*. 2019;19(3):446. doi:10.3390/s19030446

167. Zairina E, Abramson MJ, McDonald CF, et al. Study protocol for a randomised controlled trial evaluating the efficacy of a telehealth program - management of asthma with supportive telehealth of respiratory function in pregnancy (MASTERY©). *BMC Pulm Med*. 2015;15:84. doi:10.1186/s12890-015-0082-3

168. Zairina E, Abramson MJ, McDonald CF, et al. Telehealth to improve asthma control in pregnancy: A randomized controlled trial. *Respirology*. 2016;21(5):867-874. doi:10.1111/resp.12773

169. Zemet R, Schiff E, Manovitch Z, et al. Quantitative assessment of physical activity in pregnant women with sonographic short cervix and the risk for preterm delivery: A prospective pilot study. *PLoS One*. 2018;13(6):e0198949. doi:10.1371/journal.pone.0198949

170. Zhang K, Jiang M, Ma Z. The monitoring system for pregnancy-induced hypertension based on mobile communication technology. *2015 Seventh International Conference on Advanced Computational Intelligence (ICACI)*. 2015:263-266. doi:10.1109/ICACI.2015.7184789

171. Zhao L, Wu W, Zeng X, Koehl L, Tartare G. A new method for fetal movement detection using an intelligent T-shirt embedded physiological sensors. *2015 IEEE 16th International Conference on Communication Technology (ICCT)*. 2015:563-567. doi:10.1109/ICCT.2015.7399902

172. Zhao X, Zeng X, Koehl L, Tartare G, De Jonckheere J. A wearable system for in-home and long-term assessment of fetal movement. *IRBM*. 2020;41(4):205-211. doi:10.1016/j.irbm.2019.11.003

173. Zhivolupova YA. Remote monitoring system for preeclampsia detection and control. *2019 IEEE Conference of Russian Young Researchers in Electrical and Electronic Engineering (ElConRus)*. 2019:1352-1355. doi:10.1109/EIConRus.2019.8656820

2. Cancer (n = 79)

174. Armstrong KA, Coyte PC, Bhatia RS, Semple JL. The effect of mobile app home monitoring on number of in-person visits following ambulatory surgery: Protocol for a randomized controlled trial. *JMIR Res Protoc*. 2015;4(2):e65. doi:10.2196/resprot.4352

175. Armstrong KA, Coyte PC, Brown M, Beber B, Semple JL. Effect of home monitoring via mobile app on the number of in-person visits following ambulatory surgery a randomized clinical trial. *JAMA Surg*. 2017;152(7):622-627. doi:10.1001/jamasurg.2017.0111

176. Atema V, van Leeuwen M, Oldenburg HSA, et al. Design of a randomized controlled trial of Internet-based cognitive behavioral therapy for treatment-induced menopausal symptoms in breast cancer survivors. *BMC Cancer*. 2016;16(1):920. doi:10.1186/s12885-016-2946-1

177. Bhavya G, Manjunath TN, Hegadi RS, Pushpa SK. A study on personalized early detection of breast cancer using modern technology. In: Sridhar V, Padma M, Rao K, eds. *Emerging Research in Electronics, Computer Science and Technology.* Springer; 2019:355-362. doi:10.1007/978-981-13-5802-9_33

178. Brett J, Boulton M, Watson E. Development of an e-health app to support women prescribed adjuvant endocrine therapy after treatment for breast cancer. *Patient Prefer Adherence*. 2018;12:2639-2647. doi:10.2147/PPA.S187692

179. Buscemi J, Buitrago D, Iacobelli F, et al. Feasibility of a smartphone-based pilot intervention for Hispanic breast cancer survivors: A brief report. *Transl Behav Med*. 2019;9(4):638-645. doi:10.1093/tbm/iby058

180. Cai L, Boukhechba M, Gerber MS, et al. An integrated framework for using mobile sensing to understand response to mobile interventions among breast cancer patients. *Smart Health*. 2020;15:100086. doi:10.1016/j.smhl.2019.100086

181. Chaix B, Bibault JE, Pienkowski A, et al. When chatbots meet patients: One-year prospective study of conversations between patients with breast cancer and a chatbot. *JMIR Cancer*. 2019;5(1):e12856. doi:10.2196/12856

182. Chalela P, Munoz E, Inupakutika D, et al. Improving adherence to endocrine hormonal therapy among breast cancer patients: Study protocol for a randomized controlled trial. *Contemp Clin Trials Commun*. 2018;12:109-115. doi:10.1016/j.conctc.2018.10.001

183. Champ CE, Ohri N, Klement RJ, et al. Assessing changes in the activity levels of breast cancer patients during radiation therapy. *Clin Breast Cancer*. 2018;18(1):e1-e6. doi:10.1016/j.clbc.2017.08.009

184. Cohen SA, Scherr CL, Nixon DM. An iPhone application intervention to promote surveillance among women with a BRCA mutation: Pre-intervention data. *J Genet Couns*. 2018;27(2):446-456. doi:10.1007/s10897-018-0224-x

185. Coughlin SS, Besenyi GM, Bowen D, De Leo G. Development of the Physical activity and Your Nutrition for Cancer (PYNC) smartphone app for preventing breast cancer in women. *Mhealth*. 2017;3:5. doi:10.21037/mhealth.2017.02.02

186. Cruz FOAM, Vilela RA, Ferreira EB, Melo NS, Reis PEDD. Evidence on the use of mobile apps during the treatment of breast cancer: Systematic review. *JMIR Mhealth Uhealth*. 2019;7(8):e13245. doi:10.2196/13245

187. Darlow S, Heckman C. Results from a tailored SMS and behavior-tracking pilot study on sun-safe behaviors in young women. *Health Educ Behav*. 2017;44(6):937-944. doi:10.1177/1090198117699507

188. Delrieu L, Vallance JK, Morelle M, et al. Physical activity preferences before and after participation in a 6-month physical activity intervention among women with metastatic breast cancer. *Eur J Cancer Care (Engl)*. 2020;29(1):e13169. doi:10.1111/ecc.13169

189. Delrieu L, Pialoux V, Pérol O, et al. Feasibility and health benefits of an individualized physical activity intervention in women with metastatic breast cancer: Intervention study. *JMIR Mhealth Uhealth*. 2020;8(1):e12306. doi:10.2196/12306

190. Dreher N, Hadeler EK, Hartman SJ, et al. Fitbit usage in patients with breast cancer undergoing chemotherapy. *Clin Breast Cancer*. 2019;19(6):443-449.e1. doi:10.1016/j.clbc.2019.05.005

191. Egbring M, Far E, Roos M, et al. A mobile app to stabilize daily functional activity of breast cancer patients in collaboration with the physician: A randomized controlled clinical trial. *J Med Internet Res*. 2016;18(9):e238. doi:10.2196/jmir.6414

192. Ferrante JM, Devine KA, Bator A, et al. Feasibility and potential efficacy of commercial mHealth/eHealth tools for weight loss in African American breast cancer survivors: Pilot randomized controlled trial. *Transl Behav Med*. 2020;10(4):938-948. doi:10.1093/tbm/iby124

193. Fu MR, Axelrod D, Guth A, et al. A web-and mobile-based intervention for women treated for breast cancer to manage chronic pain and symptoms related to lymphedema: Randomized clinical trial rationale and protocol. *JMIR Res Protoc*. 2016;5(1):e7. doi:10.2196/resprot.5104

194. Fu MR, Axelrod D, Guth AA, et al. mHealth self-care interventions: Managing symptoms following breast cancer treatment. *Mhealth*. 2016;2:28. doi:10.21037/mhealth.2016.07.03

195. Fu MR, Axelrod D, Guth AA, et al. Usability and feasibility of health IT interventions to enhance self-care for lymphedema symptom management in breast cancer survivors. *Internet Interv*. 2016;5:56-64. doi:10.1016/j.invent.2016.08.001

196. Gell NM, Tursi A, Grover KW, Dittus K. Female cancer survivor perspectives on remote intervention components to support physical activity maintenance. *Support Care Cancer*. 2020;28(5):2185-2194. doi:10.1007/s00520-019-05038-y

197. Geng Z, Wu F, Zhang Y, et al. Mobile physical activity intervention for breast cancer patients during chemotherapy. *Stud Health Technol Inform*. 2018;250:236. doi:10.3233/978-1-61499-872-3-236

198. Gnagnarella P, Dragà D, Baggi F, et al. Promoting weight loss through diet and exercise in overweight or obese breast cancer survivors (InForma): Study protocol for a randomized controlled trial. *Trials*. 2016;17:363. doi:10.1186/s13063-016-1487-x

199. Graetz I, Anderson JN, McKillop CN, Stepanski EJ, Paladino AJ, Tillmanns TD. Use of a web-based app to improve postoperative outcomes for patients receiving gynecological oncology care: A randomized controlled feasibility trial. *Gynecol Oncol*. 2018;150(2):311-317. doi:10.1016/j.ygyno.2018.06.007

200. Haggerty AF, Huepenbecker S, Sarwer DB, et al. The use of novel technology-based weight loss interventions for obese women with endometrial hyperplasia and cancer. *Gynecol Oncol*. 2016;140(2):239-244. doi:10.1016/j.ygyno.2015.11.033

201. Harder H, Holroyd P, Burkinshaw L, et al. A user-centred approach to developing bWell, a mobile app for arm and shoulder exercises after breast cancer treatment. *J Cancer Surviv*. 2017;11(6):732-742. doi:10.1007/s11764-017-0630-3

202. Hartman SJ, Nelson SH, Cadmus-Bertram LA, Patterson RE, Parker BA, Pierce JP. Technology- and phone-based weight loss intervention: Pilot RCT in women at elevated breast cancer risk. *Am J Prev Med*. 2016;51(5):714-721. doi:10.1016/j.amepre.2016.06.024

203. Houghton LC, Howland RE, McDonald JA. Mobilizing breast cancer prevention research through smartphone apps: A systematic review of the literature. *Front Public Health*. 2019;7:298. doi:10.3389/fpubh.2019.00298

204. Hurtado-de-Mendoza A, Cabling ML, Dilawari A, et al. Providers' perspectives on adherence to hormonal therapy in breast cancer survivors. Is there a role for the digital health feedback system? *Health Technol (Berl)*. 2019;9(2):175-184. doi:10.1007/s12553-018-0267-x

205. Kang S, Yoo S, Baek H, et al. Potentials of Smart dynamometer use for clinical and self-management of rehabilitation in breast cancer survivors: A feasibility study. *Biomed Eng Lett*. 2019;9(2):211-219. doi:10.1007/s13534-019-00101-3

206. Kikawa Y, Hatachi Y, Rumpold G, et al. Evaluation of health-related quality of life via the Computer-Based Health Evaluation System (CHES) for Japanese metastatic breast cancer patients: A single-center pilot study. *Breast Cancer*. 2019;26(2):255-259. doi:10.1007/s12282-018-0905-1

207. Kim J, Lim S, Min YH, et al. Depression screening using daily mental-health ratings from a smartphone application for breast cancer patients. *J Med Internet Res*. 2016;18(8):e216. doi:10.2196/jmir.5598

208. Kim HJ, Kim SM, Shin H, Jang JS, Kim YI, Han DH. A mobile game for patients with breast cancer for chemotherapy self-management and quality-of-life improvement: Randomized controlled trial. *J Med Internet Res*. 2018;20(10):e273. doi:10.2196/jmir.9559

209. Kokts-Porietis RL, Stone CR, Friedenreich CM, Froese A, McDonough M, McNeil J. Breast cancer survivors' perspectives on a home-based physical activity intervention utilizing wearable technology. *Support Care Cancer*. 2019;27(8):2885-2892. doi:10.1007/s00520-018-4581-7

210. Lee H, Uhm KE, Cheong IY, et al. Patient satisfaction with mobile health (mHealth) application for exercise intervention in breast cancer survivors. *J Med Syst*. 2018;42(12):254. doi:10.1007/s10916-018-1096-1

211. Lidington E, McGrath SE, Noble J, et al. Evaluating a digital tool for supporting breast cancer patients: A randomized controlled trial protocol (ADAPT). *Trials*. 2020;21(1):86. doi:10.1186/s13063-019-3971-6

212. Lozano-Lozano M, Melguizo-Rodríguez L, Fernández-Lao C, et al. Association between the use of a mobile health strategy app and biological changes in breast cancer survivors: Prospective pre-post study. *J Med Internet Res*. 2019;21(8):e15062. doi:10.2196/15062

213. Lozano-Lozano M, Cantarero-Villanueva I, Martin-Martin L, et al. A mobile system to improve quality of life via energy balance in breast cancer survivors (BENECA mHealth): Prospective test-retest quasiexperimental feasibility study. *JMIR Mhealth Uhealth*. 2019;7(6):e14136. doi:10.2196/14136

214. Lynch BM, Nguyen NH, Reeves MM, et al. Study design and methods for the ACTIVity And TEchnology (ACTIVATE) trial. *Contemp Clin Trials*. 2018;64:112-117. doi:10.1016/j.cct.2017.10.015

215. Lynch BM, Nguyen NH, Moore MM, et al. A randomized controlled trial of a wearable technology-based intervention for increasing moderate to vigorous physical activity and reducing sedentary behavior in breast cancer survivors: The ACTIVATE Trial. *Cancer*. 2019;125(16):2846-2855. doi:10.1002/cncr.32143

216. Lynch BM, Nguyen NH, Moore MM, et al. Maintenance of physical activity and sedentary behavior change, and physical activity and sedentary behavior change after an abridged intervention: Secondary outcomes from the ACTIVATE Trial. *Cancer*. 2019;125(16):2856-2860. doi:10.1002/cncr.32142

217. Lyons EJ, Baranowski T, Basen-Engquist KM, et al. Testing the effects of narrative and play on physical activity among breast cancer survivors using mobile apps: Study protocol for a randomized controlled trial. *BMC Cancer*. 2016;16:202. doi:10.1186/s12885-016-2244-y

218. Martin CL, Tate DF, Valle CG. Nonadherence to daily self-weighing and activity tracking is associated with weight fluctuations among African American breast cancer survivors. *PLoS One*. 2018;13(6):e0199751. doi:10.1371/journal.pone.0199751

219. McNeil J, Brenner DR, Stone CR, et al. Activity tracker to prescribe various exercise intensities in breast cancer survivors. *Med Sci Sports Exerc*. 2019;51(5):930-940. doi:10.1249/MSS.0000000000001890

220. Melissant HC, Verdonck-de Leeuw IM, Lissenberg-Witte BI, Konings IR, Cuijpers P, Van Uden-Kraan CF. 'Oncokompas', a web-based self-management application to support patient activation and optimal supportive care: A feasibility study among breast cancer survivors. *Acta Oncol*. 2018;57(7):924-934. doi:10.1080/0284186X.2018.1438654

221. Mendes-Santos C, Weiderpass E, Santana R, Andersson G. A guided internet-delivered individually-tailored ACT-influenced cognitive behavioural intervention to improve psychosocial outcomes in breast cancer survivors (iNNOVBC): Study protocol. *Internet Interv*. 2019;17:100236. doi:10.1016/j.invent.2019.01.004

222. Mougalian SS, Epstein LN, Jhaveri AP, et al. Bidirectional text messaging to monitor endocrine therapy adherence and patient-reported outcomes in breast cancer. *JCO Clin Cancer Inform*. 2017;1:1-10. doi:10.1200/cci.17.00015

223. Nápoles AM, Santoyo-Olsson J, Chacón L, Stewart AL, Dixit N, Ortiz C. Feasibility of a mobile phone app and telephone coaching survivorship care planning program among Spanish-speaking breast cancer survivors. *JMIR Cancer*. 2019;5(2):e13543. doi:10.2196/13543

224. Nyrop KA, Deal AM, Choi SK, et al. Measuring and understanding adherence in a home-based exercise intervention during chemotherapy for early breast cancer. *Breast Cancer Res Treat*. 2018;168(1):43-55. doi:10.1007/s10549-017-4565-1

225. Ollero J, Moral-Munoz JA, Rojas I, Banos O. Mobile health system for evaluation of breast cancer patients during treatment and recovery phases. *International Conference on Bioinformatics and Biomedical Engineering.* 2017:653-664. doi:10.1007/978-3-319-56154-7_58

226. Paladino AJ, Anderson JN, Krukowski RA, et al. THRIVE study protocol: A randomized controlled trial evaluating a web-based app and tailored messages to improve adherence to adjuvant endocrine therapy among women with breast cancer. *BMC Health Serv Res*. 2019;19(1):977. doi:10.1186/s12913-019-4588-x

227. Phillips SM, Conroy DE, Keadle SK, et al. Breast cancer survivors' preferences for technology-supported exercise interventions. *Support Care Cancer*. 2017;25(10):3243-3252. doi:10.1007/s00520-017-3735-3

228. Phillips SM, Collins LM, Penedo FJ, et al. Optimization of a technology-supported physical activity intervention for breast cancer survivors: Fit2Thrive study protocol. *Contemp Clin Trials*. 2018;66:9-19. doi:10.1016/j.cct.2018.01.001

229. Phillips SM, Courneya KS, Welch WA, et al. Breast cancer survivors' preferences for mHealth physical activity interventions: Findings from a mixed methods study. *J Cancer Surviv*. 2019;13(2):292-305. doi:10.1007/s11764-019-00751-3

230. Pope Z, Lee JE, Zeng N, Lee HY, Gao Z. Feasibility of smartphone application and social media intervention on breast cancer survivors' health outcomes. *Transl Behav Med*. 2019;9(1):11-22. doi:10.1093/tbm/iby002

231. Pope ZC, Zeng N, Zhang R, Lee HY, Gao Z. Effectiveness of combined smartwatch and social media intervention on breast cancer survivor health outcomes: A 10-week pilot randomized trial. *J Clin Med*. 2018;7(6):140. doi:10.3390/jcm7060140

232. Quintiliani LM, Mann DM, Puputti M, Quinn E, Bowen DJ. Pilot and feasibility test of a mobile health-supported behavioral counseling intervention for weight management among breast cancer survivors. *JMIR Cancer*. 2016;2(1):e4. doi:10.2196/cancer.5305

233. Quintiliani LM, Foster M, Oshry LJ. Preferences of mHealth app features for weight management among breast cancer survivors from underserved populations. *Psychooncology*. 2019;28(10):2101-2104. doi:10.1002/pon.5190

234. Ritvo P, Obadia M, Santa Mina D, et al. Smartphone-enabled health coaching intervention (iMOVE) to promote long-term maintenance of physical activity in breast cancer survivors: Protocol for a feasibility pilot randomized controlled trial. *JMIR Res Protoc*. 2017;6(8):e165. doi:10.2196/resprot.6615

235. Rossi A, Frechette L, Miller D, et al. Acceptability and feasibility of a Fitbit physical activity monitor for endometrial cancer survivors. *Gynecol Oncol*. 2018;149(3):470-475. doi:10.1016/j.ygyno.2018.04.560

236. Scherr CL, Feuston JL, Nixon DM, Cohen SA. A two-phase approach to developing SNAP: An iPhone application to support appointment scheduling and management for women with a BRCA mutation. *J Genet Couns*. 2018;27(2):439-445. doi:10.1007/s10897-018-0222-z

237. Short CE, Rebar A, James EL, et al. How do different delivery schedules of tailored web-based physical activity advice for breast cancer survivors influence intervention use and efficacy? *J Cancer Surviv*. 2017;11(1):80-91. doi:10.1007/s11764-016-0565-0

238. Singh B, Spence RR, Sandler CX, Tanner J, Hayes SC. Feasibility and effect of a physical activity counselling session with or without provision of an activity tracker on maintenance of physical activity in women with breast cancer — A randomised controlled trial. *J Sci Med Sport*. 2020;23(3):283-290. doi:10.1016/j.jsams.2019.09.019

239. Solk P, Gavin K, Fanning J, et al. Feasibility and acceptability of intensive longitudinal data collection of activity and patient-reported outcomes during chemotherapy for breast cancer. *Qual Life Res*. 2019;28(12):3333-3346. doi:10.1007/s11136-019-02278-7

240. Stangl S, Haas K, Eichner FA, et al. Development and proof-of-concept of a multicenter, patient-centered cancer registry for breast cancer patients with metastatic disease - the “Breast cancer care for patients with metastatic disease” (BRE-4-MED) registry. *Pilot Feasibility Stud*. 2020;6:11. doi:10.1186/s40814-019-0541-3

241. Triberti S, Savioni L, Sebri V, Pravettoni G. eHealth for improving quality of life in breast cancer patients: A systematic review. *Cancer Treat Rev*. 2019;74:1-14. doi:10.1016/j.ctrv.2019.01.003

242. Uhm KE, Yoo JS, Chung SH, et al. Effects of exercise intervention in breast cancer patients: is mobile health (mHealth) with pedometer more effective than conventional program using brochure? *Breast Cancer Res Treat*. 2017;161(3):443-452. doi:10.1007/s10549-016-4065-8

243. Valle CG, Deal AM, Tate DF. Preventing weight gain in African American breast cancer survivors using smart scales and activity trackers: a randomized controlled pilot study. *J Cancer Surviv*. 2017;11(1):133-148. doi:10.1007/s11764-016-0571-2

244. van den Berg SW, Gielissen MFM, Custers JAE, van der Graaf WTA, Ottevanger PB, Prins JB. BREATH: Web-based self-management for psychological adjustment after primary breast cancer-Results of a multicenter randomized controlled trial. *J Clin Oncol*. 2015;33(25):2763-2771. doi:10.1200/JCO.2013.54.9386

245. van Helmondt SJ, van der Lee ML, van Woezik RAM, Lodder P, de Vries J. No effect of CBT-based online self-help training to reduce fear of cancer recurrence: First results of the CAREST multicenter randomized controlled trial. *Psychooncology*. 2020;29(1):86-97. doi:10.1002/pon.5233

246. Vijayasarveswari V, Khatun S, Fakir MM, Jusoh M, Ali S. UWB based low-cost and non-invasive practical breast cancer early detection. *AIP Conference Proceedings*. 2017;1808(1):020060. doi:10.1063/1.4975293

247. Wagoner CW, Choi SK, Deal AM, et al. Establishing physical activity in breast cancer: Self-report versus activity tracker. *Breast Cancer Res Treat*. 2019;176(2):395-400. doi:10.1007/s10549-019-05263-3

248. Wright AA, Raman N, Staples P, et al. The HOPE Pilot Study: Harnessing patient-reported outcomes and biometric data to enhance cancer care. *JCO Clin Cancer Informatics*. 2018;2:1-12. doi:10.1200/cci.17.00149

249. Wu HS, Gal R, van Sleeuwen NC, et al. Breast cancer survivors’ experiences with an activity tracker integrated into a supervised exercise program: Qualitative study. *JMIR Mhealth Uhealth*. 2019;7(2):e10820. doi:10.2196/10820

250. Zhu J, Ebert L, Liu X, Chan SW. A mobile application of breast cancer e-support program versus routine care in the treatment of Chinese women with breast cancer undergoing chemotherapy: Study protocol for a randomized controlled trial. *BMC Cancer*. 2017;17(1):291. doi:10.1186/s12885-017-3276-7

251. Zhu J, Ebert L, Xue Z, Shen Q, Chan SW. Development of a mobile application of breast cancer e-Support program for women with breast cancer undergoing chemotherapy. *Technol Health Care*. 2017;25(2):377-382. doi:10.3233/THC-161292

252. Zhu J, Ebert L, Liu X, Wei D, Chan SW. Mobile breast cancer e-support program for Chinese women with breast cancer undergoing chemotherapy (Part 2): Multicenter randomized controlled trial. *JMIR Mhealth Uhealth*. 2018;6(4):e104. doi:10.2196/mhealth.9438

3. Lifestyle (n = 58)

* 253. Abrantes AM, Blevins CE, Battle CL, Read JP, Gordon AL, Stein MD. Developing a Fitbit-supported lifestyle physical activity intervention for depressed alcohol dependent women. *J Subst Abuse Treat*. 2017;80:88-97. doi:10.1016/j.jsat.2017.07.006

254. Arigo D. Promoting physical activity among women using wearable technology and online social connectivity: A feasibility study. *Health Psychol Behav Med*. 2015;3(1):391-409. doi:10.1080/21642850.2015.1118350

255. Armin J, Johnson T, Hingle M, Giacobbi P Jr, Gordon JS. Development of a multi-behavioral mHealth app for women smokers. *J Health Commun*. 2017;22(2):153-162. doi:10.1080/10810730.2016.1256454

256. Blackshear TB, Seyfried L. Does education close the Black-White physical activity and obesity gaps? *J Am Coll Health*. 2021;69(2):222-226. doi:10.1080/07448481.2019.1657122

257. Buchan K, Morgan HM. Using the Onitor® Track for weight loss: A mixed methods study among overweight and obese women. *Health Informatics J*. 2020;26(3):1841-1865. doi:10.1177/1460458219890790

258. Buchholz SW, Wilbur JE, Halloway S, et al. Study protocol for a sequential multiple assignment randomized trial (SMART) to improve physical activity in employed women. *Contemp Clin Trials*. 2020;89:105921. doi:10.1016/j.cct.2019.105921

259. Cadmus-Bertram LA, Marcus BH, Patterson RE, Parker BA, Morey BL. Randomized trial of a Fitbit-based physical activity intervention for women. *Am J Prev Med*. 2015;49(3):414-418. doi:10.1016/j.amepre.2015.01.020

260. Cadmus-Bertram L, Marcus BH, Patterson RE, Parker BA, Morey BL. Use of the Fitbit to measure adherence to a physical activity intervention among overweight or obese, postmenopausal women: Self-monitoring trajectory during 16 weeks. *JMIR Mhealth Uhealth*. 2015;3(4):e96. doi:10.2196/mhealth.4229

* 261. Castner J, Mammen MJ, Jungquist CR, et al. Validation of fitness tracker for sleep measures in women with asthma. *J Asthma*. 2019;56(7):719-730. doi:10.1080/02770903.2018.1490753

* 262. Castner J, Jungquist CR, Mammen MJ, Pender JJ, Licata O, Sethi S. Prediction model development of women's daily asthma control using fitness tracker sleep disruption. *Heart Lung*. 2020;49(5):548-555. doi:10.1016/j.hrtlng.2020.01.013

263. Choi J, Fukuoka Y. Does having a buddy help women with young children increase physical activity? Lessons learned from a pilot study. *Women Health*. 2019;59(2):115-131. doi:10.1080/03630242.2018.1434588

* 264. Comulada WS, Swendeman D, Koussa MK, et al. Adherence to self-monitoring healthy lifestyle behaviours through mobile phone-based ecological momentary assessments and photographic food records over 6 months in mostly ethnic minority mothers. *Public Health Nutr*. 2018;21(4):679-688. doi:10.1017/S1368980017003044

265. de Zambotti M, Claudatos S, Inkelis S, Colrain IM, Baker FC. Evaluation of a consumer fitness-tracking device to assess sleep in adults. *Chronobiol Int*. 2015;32(7):1024-1028. doi:10.3109/07420528.2015.1054395

266. Depper A, Howe PD. Are we fit yet? English adolescent girls' experiences of health and fitness apps. *Health Sociol Rev*. 2017;26(1):98-112. doi:10.1080/14461242.2016.1196599

* 267. Ehlers DK, Huberty J, Buman M, Hooker S, Todd M, De Vreede GJ. A novel inexpensive use of smartphone technology for ecological momentary assessment in middle-aged women. *J Phys Act Health*. 2016;13(3):262-268. doi:10.1123/jpah.2015-0059

268. Ellis K, Kerr J, Godbole S, Staudenmayer J, Lanckriet G. Hip and wrist accelerometer algorithms for free-living behavior classification. *Med Sci Sports Exerc*. 2016;48(5):933-940. doi:10.1249/MSS.0000000000000840

* 269. Farooqi N, Slinde F, Carlsson M, Håglin L, Sandström T. Predicting energy requirement with pedometer-determined physical-activity level in women with chronic obstructive pulmonary disease. *Int J Chron Obstruct Pulmon Dis*. 2015;10:1129-1137. doi:10.2147/COPD.S80616

270. Finkelstein J, Bedra M, Li X, Wood J, Ouyang P. Mobile app to reduce inactivity in sedentary overweight women. *Stud Health Technol Inform*. 2015;216:89-92. doi:10.3233/978-1-61499-564-7-89

* 271. Fortier MS, Guerin E, Williams T, Strachan S. Should I exercise or sleep to feel better? A daily analysis with physically active working mothers. *Ment Health Phys Act*. 2015;8:56-61. doi:10.1016/j.mhpa.2015.03.001

272. Fortune E, Mundell B, Amin S, Kaufman K. A pilot study of physical activity and sedentary behavior distribution patterns in older women. *Gait Posture*. 2017;57:74-79. doi:10.1016/j.gaitpost.2017.05.014

273. Fukuoka Y, Lindgren TG, Mintz YD, Hooper J, Aswani A. Applying natural language processing to understand motivational profiles for maintaining physical activity after a mobile app and accelerometer-based intervention: The mPED randomized controlled trial. *JMIR Mhealth Uhealth*. 2018;6(6):e10042. doi:10.2196/10042

274. Fukuoka Y, Haskell W, Lin F, Vittinghoff E. Short- and long-term effects of a mobile phone app in conjunction with brief in-person counseling on physical activity among physically inactive women: The mPED randomized clinical trial. *JAMA Netw Open*. 2019;2(5):e194281. doi:10.1001/jamanetworkopen.2019.4281

275. Giacobbi P Jr, Hingle M, Johnson T, Cunningham JK, Armin J, Gordon JS. See Me Smoke-Free: Protocol for a research study to develop and test the feasibility of an mHealth app for women to address smoking, diet, and physical activity. *JMIR Res Protoc*. 2016;5(1):e12. doi:10.2196/resprot.5126

276. Griffin JB, Struempler B, Funderburk K, Parmer SM, Tran C, Wadsworth DD. My Quest, an intervention using text messaging to improve dietary and physical activity behaviors and promote weight loss in low-income women. *J Nutr Educ Behav*. 2018;50(1):11-18.e1. doi:10.1016/j.jneb.2017.09.007

277. Grossman JA, Arigo D, Bachman JL. Meaningful weight loss in obese postmenopausal women: A pilot study of high-intensity interval training and wearable technology. *Menopause*. 2018;25(4):465-470. doi:10.1097/GME.0000000000001013

278. Haney AC. *Young Female College Millennials' Intent for Behavior Change with Wearable Fitness Technology* [Doctoral Dissertation]. Walden University; 2018.

* 279. Harris BS, Melton B, Bland H, Carpentier A, Gonzales J, Catenacci K. Enhancing psychosocial constructs associated with technology-based physical activity: A randomized trial among African American women. *Am J Health Educ*. 2018;49(2):74-85. doi:10.1080/19325037.2017.1414642

280. Hasan H, Attlee A, Jan Bin Jan Mohamed H, Aris N, Bin Wan Muda WAM. Counting footsteps with a pedometer to improve HMW adiponectin and metabolic syndrome among young female adults in the United Arab Emirates. *J Obes*. 2018;2018:1597840. doi:10.1155/2018/1597840

281. Hernández-Reyes A, Cámara-Martos F, Molina Recio G, Molina-Luque R, Romero-Saldaña M, Moreno Rojas R. Push notifications from a mobile app to improve the body composition of overweight or obese women: Randomized controlled trial. *JMIR Mhealth Uhealth*. 2020;8(2):e13747. doi:10.2196/13747

282. Héroux M, Watt M, McGuire KA, Berardi JM. A personalized, multi-platform nutrition, exercise, and lifestyle coaching program: A pilot in women. *Internet Interv*. 2017;7:16-22. doi:10.1016/j.invent.2016.12.002

283. Huberty J, Ehlers DK, Kurka J, Ainsworth B, Buman M. Feasibility of three wearable sensors for 24 hour monitoring in middle-aged women. *BMC Womens Health*. 2015;15:55. doi:10.1186/s12905-015-0212-3

284. Hutchesson MJ, Callister R, Morgan PJ, et al. A targeted and tailored eHealth weight loss program for young women: The Be Positive Be Health*e* randomized controlled trial. *Healthcare (Basel)*. 2018;6(2):39. doi:10.3390/healthcare6020039

285. James DCS, Harville C II. Smartphone usage, social media engagement, and willingness to participate in mHealth weight management research among African American women. *Health Educ Behav*. 2018;45(3):315-322. doi:10.1177/1090198117714020

* 286. Jang SI, Lee M, Han J, et al. A study of skin characteristics with long-term sleep restriction in Korean women in their 40s. *Skin Res Technol*. 2020;26(2):193-199. doi:10.1111/srt.12797

287. Joseph RP, Keller C, Adams MA, Ainsworth BE. Print versus a culturally-relevant Facebook and text message delivered intervention to promote physical activity in African American women: A randomized pilot trial. *BMC Womens Health*. 2015;15:30. doi:10.1186/s12905-015-0186-1

288. Kinsey AW, Whipple M, Reid L, Affuso O. Formative assessment: Design of a web-connected sedentary behavior intervention for females. *JMIR Hum Factors*. 2017;4(4):e28. doi:10.2196/humanfactors.7670

289. Kitagawa T, Higuchi Y, Todo E, Ueda T, Ando S, Murakami T. Tailored feedback reduced prolonged sitting time and improved the health of housewives: A single-blind randomized controlled pilot study. *Women Health*. 2020;60(2):212-223. doi:10.1080/03630242.2019.1616043

290. Lanpher MG, Askew S, Bennett GG. Health literacy and weight change in a digital health intervention for women: A randomized controlled trial in primary care practice. *J Health Commun*. 2016;21(Sup1):34-42. doi:10.1080/10810730.2015.1131773

291. Leonard NR, Silverman M, Sherpa DP, et al. Mobile health technology using a wearable sensorband for female college students with problem drinking: An acceptability and feasibility study. *JMIR Mhealth Uhealth*. 2017;5(7):e90. doi:10.2196/mhealth.7399

292. Lindgren T, Hooper J, Fukuoka Y. Perceptions and experiences of women participating in a digital technology-based physical activity intervention (the mPED Trial): Qualitative study. *JMIR Public Health Surveill*. 2019;5(4):e13570. doi:10.2196/13570

293. Mann D, Riddell L, Lim K, et al. Mobile phone app aimed at improving iron intake and bioavailability in premenopausal women: A qualitative evaluation. *JMIR Mhealth Uhealth*. 2015;3(3):e92. doi:10.2196/mhealth.4300

* 294. Materia FT, Smyth JM, Heron KE, et al. Preconceptional health behavior change in women with overweight and obesity: Prototype for SMART strong healthy women intervention. *Mhealth*. 2018;4:24. doi:10.21037/mhealth.2018.06.06

295. Maxwell H, O’Shea M, Stronach M, Pearce S. Empowerment through digital health trackers: An exploration of Indigenous Australian women and physical activity in leisure settings. *Ann Leis Res*. 2021;24(1):150-167. doi:10.1080/11745398.2019.1674677

296. Memon AR, Masood T, Awan WA, Waqas A. The effectiveness of an incentivized physical activity programme (Active Student) among female medical students in Pakistan: A randomized controlled trial. *J Pak Med Assoc*. 2018;68(10):1438-1445.

297. Myers A, Gibbons C, Butler E, et al. A novel integrative procedure for identifying and integrating three-dimensions of objectively measured free-living sedentary behaviour. *BMC Public Health*. 2017;17(1):979. doi:10.1186/s12889-017-4994-0

298. Ranzenhofer LM, Engel SG, Crosby RD, et al. Real-time assessment of heart rate variability and loss of control eating in adolescent girls: A pilot study. *Int J Eat Disord*. 2016;49(2):197-201. doi:10.1002/eat.22464

299. Reeder B, Chung J, Lyden K, Winters J, Jankowski CM. Older women’s perceptions of wearable and smart home activity sensors. *Inform Health Soc Care*. 2020;45(1):96-109. doi:10.1080/17538157.2019.1582054

* 300. Richardson MB, Chmielewski C, Wu CYH, et al. The effect of time spent outdoors during summer on daily blood glucose and steps in women with type 2 diabetes. *J Behav Med*. 2020;43(5):783-790. doi:10.1007/s10865-019-00113-5

301. Riordan JK, Alexander S, Montgomery IS. Use of technology to increase physical activity in female veterans and soldiers aged 19-64 years. *J Am Assoc Nurse Pract*. 2019;31(10):575-582. doi:10.1097/JXX.0000000000000277

302. Sadek I, Mohktari M. Nonintrusive remote monitoring of sleep in home-based situation. *J Med Syst*. 2018;42(4):64. doi:10.1007/s10916-018-0917-6

303. Sayegh S, Van Der Walt M, Al-Kuwari M. One-year assessment of physical activity level in adult Qatari females: A pedometer-based longitudinal study. *Int J Womens Health*. 2016;8:287-293. doi:10.2147/IJWH.S99943

304. Sillice MA, Jennings E, Uebelacker LA, et al. African American women's relationship with their mobile phone, and what they want in a mobile delivered physical activity intervention: Guidance for intervention development. *Mhealth*. 2019;5:18. doi:10.21037/mhealth.2019.05.01

* 305. Staffileno BA, Tangney CC, Fogg L. Favorable outcomes using an eHealth approach to promote physical activity and nutrition among young African American women. *J Cardiovasc Nurs*. 2018;33(1):62-71. doi:10.1097/JCN.0000000000000409

306. Steinberg DM, Christy J, Batch BC, et al. Preventing weight gain improves sleep quality among Black women: Results from a RCT. *Ann Behav Med*. 2017;51(4):555-566. doi:10.1007/s12160-017-9879-z

* 307. van Dijk MR, Oostingh EC, Koster MPH, Willemsen SP, Laven JSE, Steegers-Theunissen RPM. The use of the mHealth program Smarter Pregnancy in preconception care: Rationale, study design and data collection of a randomized controlled trial. *BMC Pregnancy Childbirth*. 2017;17(1):46. doi:10.1186/s12884-017-1228-5

308. Wallbank G, Sherrington C, Canning CG, et al. Active women over 50: Study protocol for RCT of a low-dose information and support program to promote physical activity behaviour change. *BMC Public Health*. 2019;19(1):1225. doi:10.1186/s12889-019-7514-6

309. West DS, Stansbury M, Krukowski RA, Harvey J. Enhancing group-based internet obesity treatment: A pilot RCT comparing video and text-based chat. *Obes Sci Pract*. 2019;5(6):513-520. doi:10.1002/osp4.371

310. Zhang J, Jemmott JB III. Mobile app-based small-group physical activity intervention for young African American women: A pilot randomized controlled trial. *Prev Sci*. 2019;20(6):863-872. doi:10.1007/s11121-019-01006-4

4. Menstrual, Sexual, and Reproductive Health (n = 49)

311. Alotaibi M, Alsinan A. A mobile polycystic ovarian syndrome management and awareness system for Gulf countries: System architecture. *2016 SAI Computing Conference (SAI)*. 2016:1164-1167. doi:10.1109/SAI.2016.7556124

312. Alvergne A, Vlajic Wheeler M, Hogqvist Tabor V. Do sexually transmitted infections exacerbate negative premenstrual symptoms? Insights from digital health. *Evol Med Public Health*. 2018;2018(1):138-150. doi:10.1093/emph/eoy018

313. Berglund Scherwitzl E, Lindén Hirschberg A, Scherwitzl R. Identification and prediction of the fertile window using NaturalCycles. *Eur J Contracept Reprod Health Care*. 2015;20(5):403-408. doi:10.3109/13625187.2014.988210

314. Berglund Scherwitzl E, Lundberg O, Kopp Kallner H, et al. Short- and long-term effect of contraceptive methods on fecundity. *Eur J Contracept Reprod Health Care*. 2019;24(4):260-265. doi:10.1080/13625187.2019.1621999

315. Blödt S, Pach D, von Eisenhart-Rothe S, et al. Effectiveness of app-based self-acupressure for women with menstrual pain compared to usual care: A randomized pragmatic trial. *Am J Obstet Gynecol*. 2018;218(2):227.e1-227.e9. doi:10.1016/j.ajog.2017.11.570

316. Boyle JA, Xu R, Gilbert E, et al. Ask PCOS: Identifying need to inform evidence-based app development for polycystic ovary syndrome. *Semin Reprod Med*. 2018;36(1):59-65. doi:10.1055/s-0038-1667187

317. Bretschneider RA. A goal- and context-driven approach in mobile period tracking applications. *Universal Access in Human-Computer Interaction. Access to Learning, Health and Well-being.* 2015;9177:279-287. doi:10.1007/978-3-319-20684-4_27

* 318. Brody C, Tuot S, Chhoun P, Swendenman D, Kaplan KC, Yi S. Mobile Link - a theory-based messaging intervention for improving sexual and reproductive health of female entertainment workers in Cambodia: Study protocol of a randomized controlled trial. *Trials*. 2018;19(1):235. doi:10.1186/s13063-018-2614-7

319. Dietrich JE, Yee DL, Santos XM, et al. Assessment of an electronic intervention in young women with heavy menstrual bleeding. *J Pediatr Adolesc Gynecol*. 2017;30(2):243-246. doi:10.1016/j.jpag.2016.10.006

320. Drusany Starič K, Trajkovik V, Belani H, Vitagliano A, Bukovec P. Smart phone applications for self-monitoring of the menstrual cycle: A review and content analysis. *Clin Exp Obstet Gynecol*. 2019;46(5):731-735. doi:10.12891/ceog4830.2019

321. Duane M, Contreras A, Jensen ET, White A. The performance of fertility awareness-based method apps marketed to avoid pregnancy. *J Am Board Fam Med*. 2016;29(4):508-511. doi:10.3122/jabfm.2016.04.160022

* 322. Ernst LL, Harden CL, Pennell PB, et al. Medication adherence in women with epilepsy who are planning pregnancy. *Epilepsia*. 2016;57(12):2039-2044. doi:10.1111/epi.13586

323. Eschler J, Menking A, Fox S, Backonja U. defining menstrual literacy with the aim of evaluating mobile menstrual tracking applications. *Comput Inform Nurs*. 2019;37(12):638-646. doi:10.1097/CIN.0000000000000559

324. Faust L, Bradley D, Landau E, et al. Findings from a mobile application–based cohort are consistent with established knowledge of the menstrual cycle, fertile window, and conception. *Fertil Steril*. 2019;112(3):450-457.e3. doi:10.1016/j.fertnstert.2019.05.008

325. Fowler LR, Gillard C, Morain SR. Readability and accessibility of terms of service and privacy policies for menstruation-tracking smartphone applications. *Health Promot Pract*. 2020;21(5):679-683. doi:10.1177/1524839919899924

326. Gambier-Ross K, McLernon DJ, Morgan HM. A mixed methods exploratory study of women's relationships with and uses of fertility tracking apps. *Digit Health*. 2018;4:2055207618785077. doi:10.1177/2055207618785077

* 327. Gaskins AJ, Hart JE. The use of personal and indoor air pollution monitors in reproductive epidemiology studies. *Paediatr Perinat Epidemiol*. 2020;34(5):513-521. doi:10.1111/ppe.12599

328. Goodale BM, Shilaih M, Falco L, Dammeier F, Hamvas G, Leeners B. Wearable sensors reveal menses-driven changes in physiology and enable prediction of the fertile window: Observational study. *J Med Internet Res*. 2019;21(4):e13404. doi:10.2196/13404

329. Haile LT, Fultz HM, Simmons RG, Shelus V. Market-testing a smartphone application for family planning: Assessing potential of the CycleBeads app in seven countries through digital monitoring. *Mhealth*. 2018;4:27. doi:10.21037/mhealth.2018.06.07

330. Hutcherson TC, Cieri-Hutcherson NE, Donnelly PJ Jr, Feneziani ML, Grisanti KMR. Evaluation of mobile applications intended to aid in conception using a systematic review framework. *Ann Pharmacother*. 2020;54(2):178-186. doi:10.1177/1060028019876890

331. Jacobson AE, Vesely SK, Haamid F, Christian-Rancy M, O'Brien SH. Mobile application vs paper pictorial blood assessment chart to track menses in young women: A randomized cross-over design. *J Pediatr Adolesc Gynecol*. 2018;31(2):84-88. doi:10.1016/j.jpag.2017.09.009

332. Johnson S, Marriott L, Zinaman M. Can apps and calendar methods predict ovulation with accuracy? *Curr Med Res Opin*. 2018;34(9):1587-1594. doi:10.1080/03007995.2018.1475348

333. Johnson S, Stanford JB, Warren G, Bond S, Bench-Capon S, Zinaman MJ. Increased likelihood of pregnancy using an app-connected ovulation test system: A randomized controlled trial. *J Womens Health (Larchmt)*. 2020;29(1):84-90. doi:10.1089/jwh.2019.7850

334. Krauskopf PB. Preconception Care Quick Reference and Clue Period Tracker mobile apps. *J Nurse Pract*. 2019;15(7):526-527. doi:10.1016/j.nurpra.2019.04.005

335. Lerma K, Reyes G, Tiwari S, Tewari A, Hastings C, Blumenthal PD. Acceptability of a text message-based fertility awareness application for family planning in Lucknow, India. *Int J Gynaecol Obstet*. 2018;142(1):104-107. doi:10.1002/ijgo.12488

336. Levy J, Romo-Avilés N. "A good little tool to get to know yourself a bit better": A qualitative study on users' experiences of app-supported menstrual tracking in Europe. *BMC Public Health*. 2019;19(1):1213. doi:10.1186/s12889-019-7549-8

337. Liu B, Shi S, Wu Y, et al. Predicting pregnancy using large-scale data from a women's health tracking mobile application. *The World Wide Web Conference*. 2019:2999-3005. doi:10.1145/3308558.3313512

338. Luo L, She X, Cao J, Zhang Y, Li Y, Song PXK. Detection and prediction of ovulation from body temperature measured by an in-ear wearable thermometer. *IEEE Trans Biomed Eng*. 2020;67(2):512-522. doi:10.1109/TBME.2019.2916823

339. Maijala A, Kinnunen H, Koskimäki H, Jämsä T, Kangas M. Nocturnal finger skin temperature in menstrual cycle tracking: Ambulatory pilot study using a wearable Oura ring. *BMC Womens Health*. 2019;19(1):150. doi:10.1186/s12905-019-0844-9

* 94. Mallya R, Kothari S. Organizational structure for woman's health monitoring system using OMAS. *2017 Conference on Information and Communication Technology (CICT)*. 2017:1-6. doi:10.1109/INFOCOMTECH.2017.8340587

340. Matías-García PR, Martinez-Hurtado JL, Beckley A, Schmidmayr M, Seifert-Klauss V. Hormonal smartphone diagnostics. *Methods Mol Biol*. 2018;1735:505-515. doi:10.1007/978-1-4939-7614-0_38

341. Moglia ML, Nguyen HV, Chyjek K, Chen KT, Castaño PM. Evaluation of smartphone menstrual cycle tracking applications using an adapted APPLICATIONS scoring system. *Obstet Gynecol*. 2016;127(6):1153-1160. doi:10.1097/AOG.0000000000001444

* 342. Nwolise CH, Carey N, Shawe J. Exploring the acceptability and feasibility of a preconception and diabetes information app for women with pregestational diabetes: A mixed-methods study protocol. *Digit Health*. 2017;3:2055207617726418. doi:10.1177/2055207617726418

343. Potluri V, Kathiresan PS, Kandula H, et al. An inexpensive smartphone-based device for point-of-care ovulation testing. *Lab Chip*. 2019;19(1):59-67. doi:10.1039/c8lc00792f

* 129. Santur Y, Santur SG, Karaköse M. Architecture and implementation of a smart-pregnancy monitoring system using web-based application. *Expert Syst*. 2020;37(1):e12379. doi:10.1111/exsy.12379

344. Senette C, Buzzi MC, Paratore MT, Trujillo A. Persuasive design of a mobile coaching app to encourage a healthy lifestyle during menopause. *Proceedings of the 17th International Conference on Mobile and Ubiquitous Multimedia*. 2018:47-58. doi:10.1145/3282894.3282899

345. Shelus V, Ashcroft N, Burgess S, Giuffrida M, Jennings V. Preventing pregnancy in Kenya through distribution and use of the CycleBeads mobile application. *Int Perspect Sex Reprod Health*. 2017;43(3):131-141. doi:10.1363/43e4617

346. Shilaih M, Clerck V, Falco L, Kübler F, Leeners B. Pulse rate measurement during sleep using wearable sensors, and its correlation with the menstrual cycle phases, a prospective observational study. *Sci Rep*. 2017;7(1):1294. doi:10.1038/s41598-017-01433-9

347. Shilaih M, Goodale BM, Falco L, Kübler F, De Clerck V, Leeners B. Modern fertility awareness methods: Wrist wearables capture the changes in temperature associated with the menstrual cycle. *Biosci Rep*. 2018;38(6):BSR20171279. doi:10.1042/BSR20171279

348. Shopov M, Kakanakova I, Kakanakov N, Mateev B. An mHealth application for female fertility assistance. *2019 4th International Conference on Smart and Sustainable Technologies (SpliTech)*. 2019:1-5. doi:10.23919/SpliTech.2019.8783086

349. Sohda S, Suzuki K, Igari I. Relationship between the menstrual cycle and timing of ovulation revealed by new protocols: Analysis of data from a self-tracking health app. *J Med Internet Res*. 2017;19(11):e391. doi:10.2196/JMIR.7468

350. Symul L, Wac K, Hillard P, Salathé M. Assessment of menstrual health status and evolution through mobile apps for fertility awareness. *NPJ Digit Med*. 2019;2:64. doi:10.1038/s41746-019-0139-4

351. Timmons SE, Shakibnia EB, Gold MA, Garbers S. MyLARC: A theory-based interactive smartphone app to support adolescents' use of long-acting reversible contraception. *J Pediatr Adolesc Gynecol*. 2018;31(3):285-290. doi:10.1016/j.jpag.2017.11.005

352. Trent M, Thompson C, Tomaszewski K. Text messaging support for urban adolescents and young adults using injectable contraception: Outcomes of the DepoText pilot trial. *J Adolesc Health*. 2015;57(1):100-106. doi:10.1016/j.jadohealth.2015.03.008

* 307. van Dijk MR, Oostingh EC, Koster MPH, Willemsen SP, Laven JSE, Steegers-Theunissen RPM. The use of the mHealth program Smarter Pregnancy in preconception care: Rationale, study design and data collection of a randomized controlled trial. *BMC Pregnancy Childbirth*. 2017;17(1):46. doi:10.1186/s12884-017-1228-5

353. Vanya M, Jakó M, Füle G, et al. Use of infertility handling among women of reproductive age. *EHealth 360°: International Summit on eHealth*. 2017:497-501. doi:10.1007/978-3-319-49655-9_61

354. Wang J, Rogge AA, Armour M, et al. International ResearchKit app for women with menstrual pain: Development, access, and engagement. *JMIR Mhealth Uhealth*. 2020;8(2):e14661. doi:10.2196/14661

355. Wark JD, Henningham L, Gorelik A, Jayasinghe Y, Hartley S, Garland SM. Basal temperature measurement using a multi-sensor armband in Australian young women: A comparative observational study. *JMIR Mhealth Uhealth*. 2015;3(4):e94. doi:10.2196/mhealth.4263

356. Zwingerman R, Chaikof M, Jones C. A critical appraisal of fertility and menstrual tracking apps for the iPhone. *J Obstet Gynaecol Can*. 2020;42(5):583-590. doi:10.1016/j.jogc.2019.09.023

5. Chronic Conditions (n = 40)

357. Alshurafa N, Sideris C, Pourhomayoun M, Kalantarian H, Sarrafzadeh M, Eastwood JA. Remote health monitoring outcome success prediction using baseline and first month intervention data. *IEEE J Biomed Health Inform*. 2017;21(2):507-514. doi:10.1109/JBHI.2016.2518673

* 8. Ancker JS, Mauer E, Kalish RB, Vest JR, Gossey JT. Early adopters of patient-generated health data upload in an electronic patient portal. *Appl Clin Inform*. 2019;10(2):254-260. doi:10.1055/s-0039-1683987

358. Asklund I, Nyström E, Sjöström M, Umefjord G, Stenlund H, Samuelsson E. Mobile app for treatment of stress urinary incontinence: A randomized controlled trial. *Neurourol Urodyn*. 2017;36(5):1369-1376. doi:10.1002/nau.23116

359. Asklund I, Samuelsson E, Hamberg K, Umefjord G, Sjöström M. User experience of an app-based treatment for stress urinary incontinence: Qualitative interview study. *J Med Internet Res*. 2019;21(3):e11296. doi:10.2196/11296

360. Bliss DZ, Gurvich OV, Patel S, Meyer I, Richter HE. Self-management of accidental bowel leakage and interest in a supportive m-Health app among women. *Int Urogynecol J*. 2020;31(6):1133-1140. doi:10.1007/s00192-019-04192-5

* 261. Castner J, Mammen MJ, Jungquist CR, et al. Validation of fitness tracker for sleep measures in women with asthma. *J Asthma*. 2019;56(7):719-730. doi:10.1080/02770903.2018.1490753

* 262. Castner J, Jungquist CR, Mammen MJ, Pender JJ, Licata O, Sethi S. Prediction model development of women’s daily asthma control using fitness tracker sleep disruption. *Heart Lung*. 2020;49(5):548-555. doi:10.1016/j.hrtlng.2020.01.013

* 322. Ernst LL, Harden CL, Pennell PB, et al. Medication adherence in women with epilepsy who are planning pregnancy. *Epilepsia*. 2016;57(12):2039-2044. doi:10.1111/epi.13586

* 269. Farooqi N, Slinde F, Carlsson M, Håglin L, Sandström T. Predicting energy requirement with pedometer-determined physical-activity level in women with chronic obstructive pulmonary disease. *Int J Chron Obstruct Pulmon Dis*. 2015;10:1129-1137. doi:10.2147/COPD.S80616

361. Firet L, de Bree C, Verhoeks CM, Teunissen DAM, Lagro-Janssen ALM. Mixed feelings: General practitioners' attitudes towards eHealth for stress urinary incontinence - a qualitative study. *BMC Fam Pract*. 2019;20(1):21. doi:10.1186/s12875-019-0907-x

362. Goode PS, Markland AD, Echt K V., et al. A mobile telehealth program for behavioral treatment of urinary incontinence in women veterans: Development and pilot evaluation of MyHealth*e*Bladder. *Neurourol Urodyn*. 2020;39(1):432-439. doi:10.1002/nau.24226

363. Hildebrand K, King-Shier K, Venturato L, Tompkins-Lane C. Will women interact with technology to understand their cardiovascular risk and potentially increase activity? *Biores Open Access*. 2019;8(1):94-100. doi:10.1089/biores.2018.0047

364. Hoffman V, Söderström L, Samuelsson E. Self-management of stress urinary incontinence via a mobile app: Two-year follow-up of a randomized controlled trial. *Acta Obstet Gynecol Scand*. 2017;96(10):1180-1187. doi:10.1111/aogs.13192

365. Jamison RN, Mei A, Edwards RR, Ross EL. Efficacy of vibrating gloves for chronic hand pain due to osteoarthritis. *Pain Med*. 2018;19(5):1044-1057. doi:10.1093/pm/pnx230

366. Joseph RP, Ainsworth BE, Vega-López S, et al. Rationale and design of Smart Walk: A randomized controlled pilot trial of a smartphone-delivered physical activity and cardiometabolic risk reduction intervention for African American women. *Contemp Clin Trials*. 2019;77:46-60. doi:10.1016/j.cct.2018.12.011

367. Kathuria-Prakash N, Moser DK, Alshurafa N, Watson K, Eastwood JA. Young African American women's participation in an m-Health study in cardiovascular risk reduction: Feasibility, benefits, and barriers. *Eur J Cardiovasc Nurs*. 2019;18(7):569-576. doi:10.1177/1474515119850009

368. Lee MH, Wu HC, Tseng CM, Ko TL, Weng TJ, Chen YF. Health education and symptom flare management using a video-based m-Health system for caring women with IC/BPS. *Urology*. 2018;119:62-69. doi:10.1016/j.urology.2018.05.027

369. Lindh A, Sjöström M, Stenlund H, Samuelsson E. Non-face-to-face treatment of stress urinary incontinence: Predictors of success after 1 year. *Int Urogynecol J*. 2016;27(12):1857-1865. doi:10.1007/s00192-016-3050-4

370. Long JE. *Mind Over Bladder: Women, Aging and Bladder Health* [Doctoral Dissertation]. University of North Carolina at Chapel Hill; 2017.

371. Loohuis AMM, Wessels NJ, Jellema P, et al. The impact of a mobile application-based treatment for urinary incontinence in adult women: Design of a mixed-methods randomized controlled trial in a primary care setting. *Neurourol Urodyn*. 2018;37(7):2167-2176. doi:10.1002/nau.23507

372. Mayo RC, Leung J. Novel wireless cardiac monitor located in the breast: Imaging appearance and function. *Breast J*. 2017;23(5):599-601. doi:10.1111/tbj.12803

373. Nes AAG, van Dulmen S, Wicksell R, Fors EA, Eide H. Analyzing change processes resulting from a smartphone maintenance intervention based on acceptance and commitment therapy for women with chronic widespread pain. *Int J Behav Med*. 2017;24(2):215-229. doi:10.1007/s12529-016-9590-7

374. Neville C, Da Costa D, Rochon M, et al. Development of the lupus interactive navigator as an empowering web-based eHealth tool to facilitate lupus management: Users perspectives on usability and acceptability. *JMIR Res Protoc*. 2016;5(2):e44. doi:10.2196/resprot.4219

* 342. Nwolise CH, Carey N, Shawe J. Exploring the acceptability and feasibility of a preconception and diabetes information app for women with pregestational diabetes: A mixed-methods study protocol. *Digit Health*. 2017;3:2055207617726418. doi:10.1177/2055207617726418

375. Nyström E, Asklund I, Sjöström M, Stenlund H, Samuelsson E. Treatment of stress urinary incontinence with a mobile app: Factors associated with success. *Int Urogynecol J*. 2018;29(9):1325-1333. doi:10.1007/s00192-017-3514-1

376. Park YJ, Lee SJ, Shin NM, et al. Application and effect of mobiletype-bone health intervention in Korean young adult women with low bone mass: A randomized control trial. *Asian Nurs Res (Korean Soc Nurs Sci)*. 2017;11(1):56-64. doi:10.1016/j.anr.2017.03.005

377. Ravn Jakobsen P, Hermann AP, Søndergaard J, Wiil UK, Clemensen J. Development of an mHealth application for women newly diagnosed with osteoporosis without preceding fractures: A participatory design approach. *Int J Environ Res Public Health*. 2018;15(2):330. doi:10.3390/ijerph15020330

378. Ravn Jakobsen P, Hermann AP, Søndergaard J, Wiil UK, Clemensen J. Help at hand: Women's experiences of using a mobile health application upon diagnosis of asymptomatic osteoporosis. *SAGE Open Med*. 2018;6:205031211807617. doi:10.1177/2050312118807617

* 300. Richardson MB, Chmielewski C, Wu CYH, et al. The effect of time spent outdoors during summer on daily blood glucose and steps in women with type 2 diabetes. *J Behav Med*. 2020;43(5):783-790. doi:10.1007/s10865-019-00113-5

379. Ryan P, Papanek P, Csuka ME, et al. Background and method of the Striving to be Strong study a RCT testing the efficacy of a m-health self-management intervention. *Contemp Clin Trials*. 2018;71:80-87. doi:10.1016/j.cct.2018.06.006

380. Ryan P, Brown RL, Csuka ME, Papanek P. Efficacy of osteoporosis prevention smartphone app. *Nurs Res*. 2020;69(1):31-41. doi:10.1097/NNR.0000000000000392

* 381. Sakakibara BM, Ross E, Arthur G, et al. Using mobile-health to connect women with cardiovascular disease and improve self-management. *Telemed J E Health*. 2017;23(3):233-239. doi:10.1089/tmj.2016.0133

382. Shelly B. Pelvic muscle exercises using a home trainer for pelvic muscle dysfunction: A case report. *Urol Nurs*. 2016;36(2):82-87. doi:10.7257/1053-816x.2016.36.2.82

383. Sjöström M, Umefjord G, Stenlund H, Carlbring P, Andersson G, Samuelsson E. Internet-based treatment of stress urinary incontinence: 1- and 2-year results of a randomized controlled trial with a focus on pelvic floor muscle training. *BJU Int*. 2015;116(6):955-964. doi:10.1111/bju.13091

384. Sjöström M, Lindholm L, Samuelsson E. Mobile app for treatment of stress urinary incontinence: A cost-effectiveness analysis. *J Med Internet Res*. 2017;19(5):e154. doi:10.2196/jmir.7383

* 305. Staffileno BA, Tangney CC, Fogg L. Favorable outcomes using an eHealth approach to promote physical activity and nutrition among young African American women. *J Cardiovasc Nurs*. 2018;33(1):62-71. doi:10.1097/JCN.0000000000000409

385. Subasinghe AK, Garland SM, Gorelik A, Tay I, Wark JD. Using mobile technology to improve bone-related lifestyle risk factors in young women with low bone mineral density: Feasibility randomized controlled trial. *JMIR Form Res*. 2019;3(1):e9435. doi:10.2196/formative.9435

386. Tay I, Garland S, Gorelik A, Wark JD. Development and testing of a mobile phone app for self-monitoring of calcium intake in young women. *JMIR Mhealth Uhealth*. 2017;5(3):e27. doi:10.2196/mhealth.5717

387. Thomas S, Yingling L, Adu-Brimpong J, et al. Mobile health technology can objectively capture physical activity (PA) targets among African-American women within resource-limited communities—the Washington, D.C. Cardiovascular Health and Needs Assessment. *J Racial Ethn Health Disparities*. 2017;4(5):876-883. doi:10.1007/s40615-016-0290-4

388. Wenger NK, Williams OO, Parashar S. SMARTWOMAN™: Feasibility assessment of a smartphone app to control cardiovascular risk factors in vulnerable diabetic women. *Clin Cardiol*. 2019;42(2):217-221. doi:10.1002/clc.23124

6. Other (n = 26)

* 253. Abrantes AM, Blevins CE, Battle CL, Read JP, Gordon AL, Stein MD. Developing a Fitbit-supported lifestyle physical activity intervention for depressed alcohol dependent women. *J Subst Abuse Treat*. 2017;80:88-97. doi:10.1016/j.jsat.2017.07.006

* 318. Brody C, Tuot S, Chhoun P, Swendenman D, Kaplan KC, Yi S. Mobile Link - a theory-based messaging intervention for improving sexual and reproductive health of female entertainment workers in Cambodia: Study protocol of a randomized controlled trial. *Trials*. 2018;19(1):235. doi:10.1186/s13063-018-2614-7

389. Burnett-Zeigler IE, Waldron EM, Hong S, Yang A, Wisner KL, Ciolino JD. Accessibility and feasibility of using technology to support mindfulness practice, reduce stress and promote long term mental health. *Complement Ther Clin Pract*. 2018;33:93-99. doi:10.1016/j.ctcp.2018.09.001

* 264. Comulada WS, Swendeman D, Koussa MK, et al. Adherence to self-monitoring healthy lifestyle behaviours through mobile phone-based ecological momentary assessments and photographic food records over 6 months in mostly ethnic minority mothers. *Public Health Nutr*. 2018;21(4):679-688. doi:10.1017/S1368980017003044

390. Costa J, Figueiredo P, Nakamura F, Rago V, Rebelo A, Brito J. Intra-individual variability of sleep and nocturnal cardiac autonomic activity in elite female soccer players during an international tournament. *PLoS One*. 2019;14(9):e0218635. doi:10.1371/journal.pone.0218635

391. Douglas A, Rotondi MA, Baker J, Jamnik VK, MacPherson AK. On-ice physical demands of world-class women's ice hockey: From training to competition. *Int J Sports Physiol Perform*. 2019;14(9):1227-1232. doi:10.1123/ijspp.2018-0571

* 267. Ehlers DK, Huberty J, Buman M, Hooker S, Todd M, De Vreede GJ. A novel inexpensive use of smartphone technology for ecological momentary assessment in middle-aged women. *J Phys Act Health*. 2016;13(3):262-268. doi:10.1123/jpah.2015-0059

392. Flatt AA, Esco MR. Evaluating individual training adaptation with smartphone-derived heart rate variability in a collegiate female soccer team. *J Strength Cond Res*. 2016;30(2):378-385. doi:10.1519/JSC.0000000000001095

* 271. Fortier MS, Guerin E, Williams T, Strachan S. Should I exercise or sleep to feel better? A daily analysis with physically active working mothers. *Ment Health Phys Act*. 2015;8:56-61. doi:10.1016/j.mhpa.2015.03.001

* 327. Gaskins AJ, Hart JE. The use of personal and indoor air pollution monitors in reproductive epidemiology studies. *Paediatr Perinat Epidemiol*. 2020;34(5):513-521. doi:10.1111/ppe.12599

* 279. Harris BS, Melton B, Bland H, Carpentier A, Gonzales J, Catenacci K. Enhancing psychosocial constructs associated with technology-based physical activity: A randomized trial among African American women. *Am J Health Educ*. 2018;49(2):74-85. doi:10.1080/19325037.2017.1414642

393. Hegarty K, Tarzia L, Murray E, et al. Protocol for a randomised controlled trial of a web-based healthy relationship tool and safety decision aid for women experiencing domestic violence (I-DECIDE). *BMC Public Health*. 2015;15:736. doi:10.1186/s12889-015-2072-z

* 286. Jang SI, Lee M, Han J, et al. A study of skin characteristics with long-term sleep restriction in Korean women in their 40s. *Skin Res Technol*. 2020;26(2):193-199. doi:10.1111/srt.12797

* 294. Materia FT, Smyth JM, Heron KE, et al. Preconceptional health behavior change in women with overweight and obesity: Prototype for SMART strong healthy women intervention. *Mhealth*. 2018;4:24. doi:10.21037/mhealth.2018.06.06

394. Menaspà MJ, Menaspà P, Clark SA, Fanchini M. Validity of the online athlete management system to assess training load. *Int J Sports Physiol Perform*. 2018;13(6):750-754. doi:10.1123/ijspp.2017-0379

395. Montoye AHK, Mitrzyk J. Validity of the Blast Athletic Performance monitor for assessing vertical jump height in female volleyball players. *Meas Phys Educ Exerc Sci*. 2019;23(2):99-109. doi:10.1080/1091367X.2018.1539739

396. Pillarisetti A, Carter E, Rajkumar S, et al. Measuring personal exposure to fine particulate matter ($PM_{2.5}$) among rural Honduran women: A field evaluation of the Ultrasonic Personal Aerosol Sampler (UPAS). *Environ Int*. 2019;123:50-53. doi:10.1016/j.envint.2018.11.014

397. Ragavan MI, Ferre V, Bair-Merritt M. Thrive: A novel health education mobile application for mothers who have experienced intimate partner violence. *Health Promot Pract*. 2020;21(2):160-164. doi:10.1177/1524839919890870

398. Relf R, Willmott A, Flint MS, Beale L, Maxwell N. Reliability of a wearable sweat rate monitor and routine sweat analysis techniques under heat stress in females. *J Therm Biol*. 2019;79:209-217. doi:10.1016/j.jtherbio.2018.12.019

* 381. Sakakibara BM, Ross E, Arthur G, et al. Using mobile-health to connect women with cardiovascular disease and improve self-management. *Telemed J E Health*. 2017;23(3):233-239. doi:10.1089/tmj.2016.0133

399. Sanders GJ, Boos B, Rhodes J, Peacock CA, Kollock RO, Scheadler CM. Variability of competition-based caloric expenditure and relative heart rates in National Collegiate Athletic Association Division I women’s basketball. *J Strength Cond Res*. 2022;36(1):162-166. doi:10.1519/JSC.0000000000003404

400. Sanders GJ, Boos B, Rhodes J, Kollock RO, Peacock CA. Competition-based heart rate, training load, and time played above 85% peak heart rate in NCAA Division I women's basketball. *J Strength Cond Res*. 2021;35(4):1095-1102. doi:10.1519/JSC.0000000000002876

401. Taylor M, Nagle EF, Goss FL, Rubinstein EN, Simonson A. Evaluating energy expenditure estimated by wearable technology during variable intensity activity on female collegiate athletes. *Int J Exerc Sci*. 2018;11(7):598-608.

402. Thompson LA, Mercado R, Martinko T, Acharya R. Novel interventions and assessments using patient portals in adolescent research: Confidential survey study. *J Med Internet Res*. 2018;20(3):e101. doi:10.2196/jmir.8340

403. Tibana RA, de Sousa NMF, Prestes J, Feito Y, Ernesto C, Voltarelli FA. Monitoring training load, well-being, heart rate variability, and competitive performance of a functional-fitness female athlete: A case study. *Sports (Basel)*. 2019;7(2):35. doi:10.3390/sports7020035

404. Wells AJ, Hoffman JR, Beyer KS, et al. Regular- and postseason comparisons of playing time and measures of running performance in NCAA Division I women soccer players. *Appl Physiol Nutr Metab*. 2015;40(9):907-917. doi:10.1139/apnm-2014-0560

7. No Specific Health Area (n = 2)

405. James DCS, Harville C, Whitehead N, Stellefson M, Dodani S, Sears C. Willingness of African American women to participate in e-Health/m-Health research. *Telemed J E Health*. 2016;22(3):191-197. doi:10.1089/tmj.2015.0071

406. Lupton D, Maslen S. The more-than-human sensorium: Sensory engagements with digital self-tracking technologies. *Senses Soc*. 2018;13(2):190-202. doi:10.1080/17458927.2018.1480177
