# Supplementary material for: Person-Generated Health Data in Women’s Health: Scoping Review
Source: J Med Internet Res. 2024 May 16;26:e53327. doi: 10.2196/53327 (PMC11140278; doi:10.2196/53327)
Supplement: Multimedia Appendix 4 [file jmir_v26i1e53327_app4.pdf]

Multimedia Appendix 4: Metrics that were collected through digital health tools

**Personal info and functions**	
	Demographics, medical history, or user profile
	Location (GPS)
	Location (self-reported)
	Time zone change
	Goal setting function
	Uploading photos/videos, including: baby bump photos, baby photos, ultrasound images, photos of food, photos of wounds
	Facial recognition
	Calendar (appointments, events, prescriptions)
	Prescription refill request
	Free text/notes/diary/voice recording
	Baby sound recorder
	Episodic audio recording – to detect sound of environment
	Audio recording function for doctor's visit
	Interactive voice messaging system
	Discussion board/online forum/social networks/direct messaging
	Message health care provider or schedule medical appointment
	Store health care provider contact information
	Ask the app specific questions
	Text the app for help/advice
**Mental health, psychosocial measures, and pain-related**	
	Mental well-being, mood tracker, or psychological status
	Happiness during pregnancy
	Worry
	Energy level/fatigue
	Enjoyment
	Anxiety
	Depression
	Mood
	Affect
	Quality of Life
	Health-related Quality of Life
	Met/unmet needs
	Stress
	Self worth
	Self esteem
	Social support
	Cognitive function
	Physical ability
	Fear of cancer recurrence
	Self regulation (EMA*)
	Experiences of racism/microaggressions
	Pain/pain severity
	Pain fear/avoidance
	Pain catastrophizing
	Pain acceptance
	Pain self management
	Pain with intercourse
	Location on body where pain is experienced
	Quality of recovery
	Prepartum depression
	Postpartum depression
	Satisfaction with care
	Self efficacy
	Tracking mindfulness practice
	Support

**Anthropometric and general body-related**
Posture (EMA*)
Self-reported barriers to reaching weight loss goals
Body mass index (BMI)
Weight/weight gain
Waist circumference
Shin circumference
Height
**Physical activity, cardiovascular, or respiratory-related**
Training load
Muscle soreness
Step count
Step cadence
Stride length
Aerobic steps
Distance travelled
Running speed
Jump height
Floors climbed/elevation
Grip strength / pressure
Grip reaction time
Grip endurance
Training load
Muscle soreness
Balance/strength
Reasons for not exercising/not meeting physical activity goals
Sedentary behaviour/time sitting
Physical activity/active minutes
Perceived physical exertion
Intensity of physical activity
Calories expended/metabolism
Metabolic equivalent rate (MET)
Blood pressure reading
Radial artery pulse wave signal
Lung function/obstruction (forced expiratory volume)
Respiratory rate
Heart rate/heart rate variability
VO2
Cardiac activity monitor
Pulse pressure
Skin perfusion
Blood oxygen saturation (oximeter)
Near body ambient temperature
Body temperature
Wrist skin temperature
Sweat measurements (electrodermal activity, volume)
Sweat rate
Sweat gland activation
Skin conductance
Sweat conductivity
ECG
**Food, diet, and other consumption-related**
Calorie counting
Diet, food diary (eating/drinking habits), or nutrition
Reasons for not meeting dietary goals
Food preferences
Food budget
Intake of vitamins and minerals (calcium, vitamin D)
Cravings
Appetite
Loss of control eating tracking
Degree of hunger before meal
Planned/unplanned meals
Alcohol consumption
Caffeine consumption

Blood alcohol level (breathalyzer)
Cost savings calculator for quitting smoking
Smoking “quit” date
Smoking behaviour/tracking
Number of cigarettes smoked
Smoking cravings
Smoking withdrawal symptoms
**Pregnancy and postpartum-related**
Trimester of pregnancy
Whether it is a first pregnancy
Pregnancy due date/milestones for fetal development tracking (eg, gestational week)
Preeclampsia symptoms
Protein changes in urine/proteinuria
Prenatal vitamins taken (folic acid)
Prenatal ultrasound
Fundal height
Estimated fetal weight
Pregnancy or birthing discomforts/symptoms
Pregnancy outcome
Neonatal outcomes
Cervical dilation
Uterine contractions
Fetal kicks/movement
Fetal ECG
CTG monitoring
Fetal heart rate/heart rate variation
Fetal anthropometrics
Embryotic fluid level
Fetal hypoxia
Baby’s sex
Birth records

Vaccination record for newborn/child
Infant mood
Postpartum events/symptoms
Postpartum depression
Number of wet/dirty diapers or bowel movements
Breastfeeding challenges, annoyances
Breastfeeding patterns/tracking
Which breast during feed
Latching/breastfeeding position
Breast milk expression time tracker/pumping record
Bottle feeding tracker
Volume of breast milk expressed
Breastfeeding locations
Sleeping patterns of newborn
Proximity to baby
Baby’s medical history
Baby’s anthropometric measurements
Baby’s mood
Baby/infant milestones
Baby’s medication
Provision and duration of kangaroo care
**Disease/condition and cancer-related**
Asthma symptoms
Asthma medications
UVR exposure
Sun protection
Duration of activities done in clear daylight (vitamin D exposure)
Blood glucose levels
HbA1c levels
Ketonuria analysis results
Proteinuria analysis results

Current medications/adherence to medications and treatment regimens
Reasons for not taking medication
Insulin dosage
Metformin dosage
Illness events
Perioperative symptoms
Incontinence
Tracking pelvic floor exercise completion
Pelvic floor muscles – maximum voluntary contraction, relaxation
Urinary incontinence, number of daily voids
Urinary incontinence, daily leaks (symptom severity)
Urinary incontinence, number of pad changes
Urinary incontinence – Quality of Life
Breast cancer detection - sensor
Cancer diagnosis
Arm mobility (following breast cancer)
Tracking upper arm and shoulder exercises for upper limb dysfunction
Arm and shoulder flexibility
Breast cancer symptom tracker
Side effects/symptoms of cancer treatment
Severity of cancer treatment side effects/symptoms
Interference of ADLs due to severity of cancer symptoms
Post surgical symptoms and severity
Lymphedema symptoms/severity
Completion of daily lymphedema exercises
Osteoarthritis pain intensity
Perceived osteoarthritis pain improvement
Pain interference with activities of daily living
Tracking wear time of osteoarthritis gloves

Tracking exercises for osteoporeosis
Symptoms of hypertension (headache, epigastric pain, nausea, vomiting, visual symptoms)
Breast abnormalities
**Menstruation, fertility, and sexual health-related**
Basal body temperature
Photo of urine dipstick
Ovulation/LH/urine test results
Saliva sample microfluidic sensor
STI symptoms
Previous STI treatments
Cervical fluid during the menstrual cycle
Cervical characteristics (open/closed, high/low)
Vaginal sensations (wetness)
Fever occurrence
Shower before sleep
Menstrual cycle tracker
Age of menarche
Menstrual cycle symptoms
Spotting between periods
Period blood volume estimation
Menstrual clots
Menstrual breakthrough bleeding/flooding
Intercourse/sexual activity
Sexual health
Sex drive
Sexuality
Pain medication taken
Number of days of sick leave taken
Self care activities
Tracking acupressure sessions
Pain during menstrual cycle
Adverse reactions to acupressure

	Emotions and mood tracking during menstrual cycle
	Hormonal contraceptive use
	Description of contraceptive use
	Pregnancy test result
	Menopausal symptoms
**Other**	
	Lab results
	Bowel movements/description
	Current activity/type of activity (EMA*)
	Symptoms following vaccination
	Reasons for not completing the challenge
	Sleep quality/measurements/satisfaction
	Sleep interruptions/sleeping irregularities
	Sleep stage
	Sleep time/duration
	Assessment of daily functional activity
	Environmental sensors (temperature, humidity, air quality, air pressure, noise, quality of potable water)
	Self reported environmental exposures
	Humidity
	Air quality
	Noise
	Quality of their potable water
	Seizures
	Current level of safety
	Current level of fear in the relationship
	How healthy she believes that her relationship is
	Danger assessment

* EMA = ecological momentary assessment
